# Lanthanum Carbonate Nanosheets Based Colloidal Hydrogel for Modulating Innate & Adaptive Tumor Immunotherapy by Augmented Cellular Pyroptosis

**DOI:** 10.1002/advs.76966

**Published:** 2026-08-03

**Authors:** Yang Hong, Haitao Wu, Weitao Wang, Xuan Zhang, Qian Chen, Yaoyu Hu, Zhengbao Zha

**Affiliations:** ^1^ School of Medicine and Health Zhengzhou Advanced Research Institute of Harbin Institute of Technology Zhengzhou China; ^2^ School of Food and Biological Engineering Hefei University of Technology Hefei China; ^3^ School of Biomedical Engineering Anhui Medical University Hefei China

**Keywords:** colloidal hydrogel, GSDME, immunotherapy, lanthanum carbonate nanosheets, pyroptosis

## Abstract

Cellular pyroptosis, a pro‐inflammatory form of programmed cell death, emerges as a promising strategy for cancer immunotherapy. However, gene methylation hampers the expression of gasdermin E (GSDME), a pivotal protein in the caspase‐3‐mediated pyroptosis pathway, across numerous malignancies, including triple‐negative breast cancer (TNBC). This study elucidated that a colloidal hydrogel (GDLC hydrogel) loaded with decitabine (DAC) and crosslinked with lanthanum carbonate nanosheets (LCNSs) and gelatin nanoparticles (Gela NPs) could instigate pyroptosis in TNBC cells, specifically 4T1 cells. Within the tumor microenvironment (TME), the synergistic interaction between DAC and La^3+^ reversed the epigenetic silencing of the GSDME gene, while concurrently activating caspase‐3, thus facilitating pyroptosis induction. Although monotherapy with La^3+^‐induced apoptosis proved insufficient in tumor suppression, the concurrent inhibition of methyltransferase via DAC markedly suppressed both primary and recurrent tumors. Moreover, the combinatory application of the GDLC hydrogel and immune checkpoint inhibitors effectively neutralized the immunosuppressive TME of TNBC, triggered an anti‐tumor immune response and engendered a sustained immune memory effect. This strategy significantly curtailed tumor recurrence and metastasis, consequently improving the survival rates of mice in the 4T1 tumor‐bearing mouse model. In conclusion, this colloidal hydrogel platform introduced an innovative paradigm for the immunotherapy of triple‐negative breast cancer.

## Graphical abstract

The Gela/decitabine/La_2_(CO_3_)_3_ colloidal (GDLC) hydrogel developed here could reverse the epigenetic silencing of the GSDME gene by the synergistic interaction between DAC and La^3+^, while concurrently activating caspase‐3, thus facilitating pyroptosis induction under the tumor microenvironment. Moreover, the combinatory application of the GDLC hydrogel and immune checkpoint inhibitors effectively triggered robust anti‐tumor innate & adaptive immune responses and engendered a sustained immune memory effect.

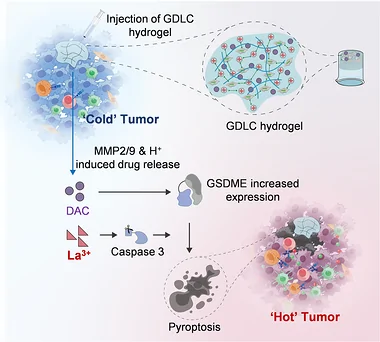

## 1 Introduction

Cellular pyroptosis, an immunogenic form of programmed cell death, has garnered significant attention in the field of cancer immunotherapy [1, 2]. Pyroptosis is distinct from non‐immunogenic apoptosis due to the formation of plasma membrane pores, subsequent membrane rupture, and cellular swelling [3]. These mechanisms ultimately lead to the release of cytoplasmic contents, including pro‐inflammatory cytokines and lactate dehydrogenase, followed by the activation of immune cells to enhance anti‐tumor immunotherapy [4]. However, the episodic silencing of pyroptosis‐interacting proteins, such as Gasdermin E (GSDME) in breast cancer and Gasdermin A/C/D (GSDMA/C/D) in gastric cancer, limits the potential applications of pyroptosis in cancer treatment [5, 6, 7]. Decitabine (DAC), a DNA methyltransferase inhibitor, has been shown to be capable of reversing pyroptosis gene silencing and enhancing Gasdermin expression, thereby making pyroptosis a feasible outcome [8, 9]. Additionally, current pyroptosis‐based cancer immunotherapy strategies still have limitations. Pyroptosis therapies alone demonstrate limited efficacy due to the suppressive immune microenvironment in tumors [10]. Although several studies have investigated the interplay between pyroptosis and immune checkpoint blockade therapy, the majority have concentrated on integrating these approaches with adaptive immunization strategies aimed at T cells. Nonetheless, these strategies are constrained by their dependence on the activation of the innate immune system [11]. T cell immunoreceptor with Ig and ITIM domains (TIGIT), a recently identified immune checkpoint present on T cells and innate immune natural killer (NK) cells, has garnered significant interest in the scientific community [12, 13, 14, 15]. Thus, blocking TIGIT with the induction of synergistic pyroptosis could potentially develop a highly effective cancer immunotherapy strategy that engages both innate and adaptive immunity at multiple levels.

Lanthanide ions (III) have attracted widespread interest in the biomedical field, with various lanthanide compounds exhibiting pharmacological properties explored in clinical investigations [16, 17, 18]. Notably, La^3+^ has been shown to inhibit the proliferation of cervical cancer cells and promote apoptosis by modulating the expression of Cyclin D1, Bcl‐2, and Caspase‐3 [19]. Lanthanum carbonate chewable tablets represent a prominent biomedical application of La^3+^, approved for managing hyperphosphatemia in dialysis patients owing to its exceptional phosphate chelation capabilities and safety profile [20]. The potential of lanthanum carbonate as a carrier for La^3+^ in cancer therapy is promising, particularly in its reactivity to acidity as a carbonate compound, enabling targeted release in the acidic tumor microenvironment (TME). Enhancements in lanthanum carbonate formulations utilizing nanotechnology are crucial to address the challenges in delivering free La^3+^ efficiently to tumor locations [21].

Colloidal hydrogels have emerged as a promising class of gel matrices with significant potential for drug delivery applications [22]. A key advantage lies in their ability to immobilize therapeutic agents via electrostatic interactions with nanoparticles. These reversible electrostatic interactions facilitate efficient, secure, and intelligent drug delivery mechanisms [23]. In this study, a colloidal hydrogel was engineered as a pyroptosis inducer to counteract the gene silencing of pyroptosis pathways, with the goal of boosting anti‐tumor immunotherapy by specifically inducing pyroptosis in breast cancer cells (Figure 1). Gelatin nanoparticles (Gela NPs) and lanthanum carbonate nanosheets (LCNSs) were initially suspended in an alkaline medium. Upon pH adjustment, electrostatic interaction prompted nanoparticle cross‐linking, forming colloidal hydrogels encapsulating DAC, termed as GDLC hydrogel. The biocompatibilities of gelatin and lanthanum carbonate ensure the biosafety of GDLC hydrogel. Gela NPs and LCNSs facilitate the precise and responsive release of DAC under acidic conditions and high levels of matrix metalloproteinase 2/9 (MMP 2/9) in the presence of La^3+^, making it suitable for the acidic TME. Within the breast cancer TME, effective DAC delivery led to the demethylation of GSDME gene. The activation of caspase‐3 mediated by La^3+^ led to the production of cleaved caspase‐3, which facilitated the synergistic induction of pyroptosis in breast cancer cells. The significantly inhibited tumor growth and activated robust anti‐tumor immune responses led to a reversal of the immunosuppressive conditions within the TME. Furthermore, the concurrent administration of TIGIT inhibitors amplified anti‐tumor immune responses, induced durable immune memory, and demonstrated potential in preventing tumor recurrence and metastasis.

**FIGURE 1:**
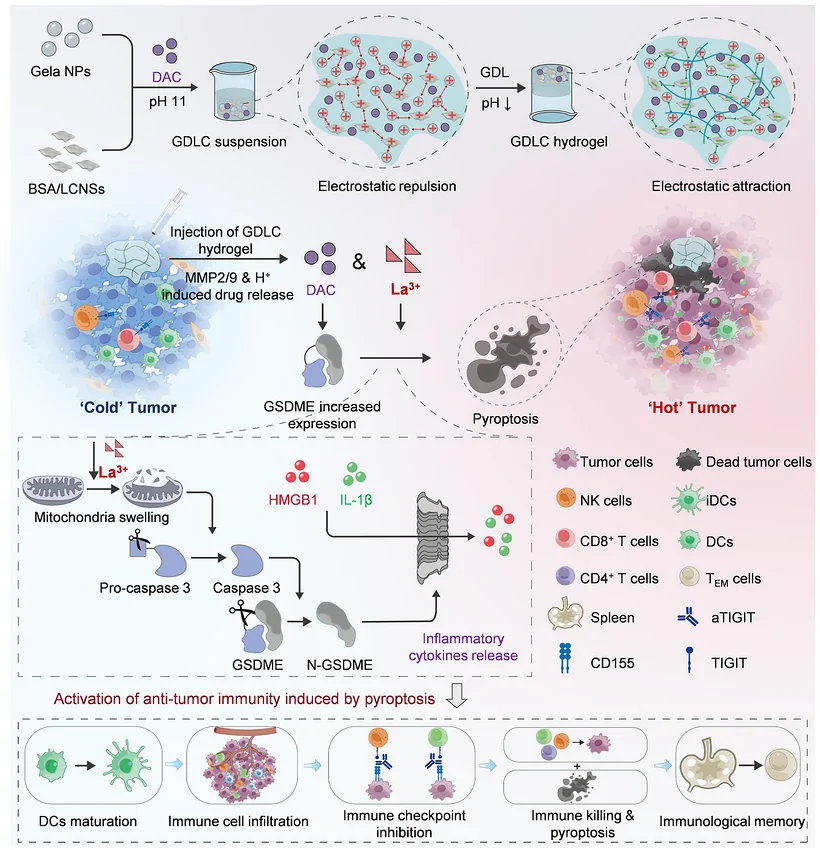
Schematic representation of GDLC hydrogel preparation and the mechanism for its anti‐tumor immune activation. A colloidal hydrogel of Gela NPs cross‐linked with LCNSs was prepared. The acidic TME caused the release of La^3+^ and DAC. The effect of DAC resulted in decreased silence of the GSDME gene in breast cancer, while La^3+^ in the microenvironment activated caspase‐3, which further triggered GSDME‐induced cellular pyroptosis. The combination of pyroptosis and anti‐TIGIT antibody (aTIGIT) led to the inhibition of tumor growth, a strong anti‐tumor immune response in mice, and the formation of a long‐term immune memory effect.

## 2 Results

### 2.1 Preparation and Characterization of BSA/LCNSs

The LCNSs were synthesized via an acousto‐chemical method, wherein La^3+^ ions were precipitated with CO_3_^2−^ under ultrasonic irradiation. Transmission electron microscopy (TEM) images and particle size distribution analysis revealed the sheet morphologies of LCNSs, with sizes ranging from 400 to 1000 nm with an average particle size of 609 nm (Figure 2a and Figure S1). Atomic force microscope (AFM) results indicated the presence of sheet‐like structures with a thickness of approximately 2 nm (Figure 2b). Energy dispersive X‐ray spectroscopy (EDS) elemental mapping confirmed the homogeneous distribution of La and O elements within the LCNSs (Figure 2c). High‐resolution transmission electron microscopy (HRTEM) images validated the crystalline structure, displaying a lattice spacing of 0.322 nm corresponding to the (220) plane (Figure 2d). X‐ray diffraction (XRD) analysis was conducted to further corroborate the crystalline structure, with characteristic diffraction peaks at 10.4, 19.7, and 27.2 corresponding to the (002), (020), and (220) planes, respectively (Figure 2e). X‐ray photoelectron spectroscopy (XPS) analysis also confirmed the presence of La^3+^ ions in the as‐prepared LCNSs (Figure 2f and Figure S2).

**FIGURE 2:**
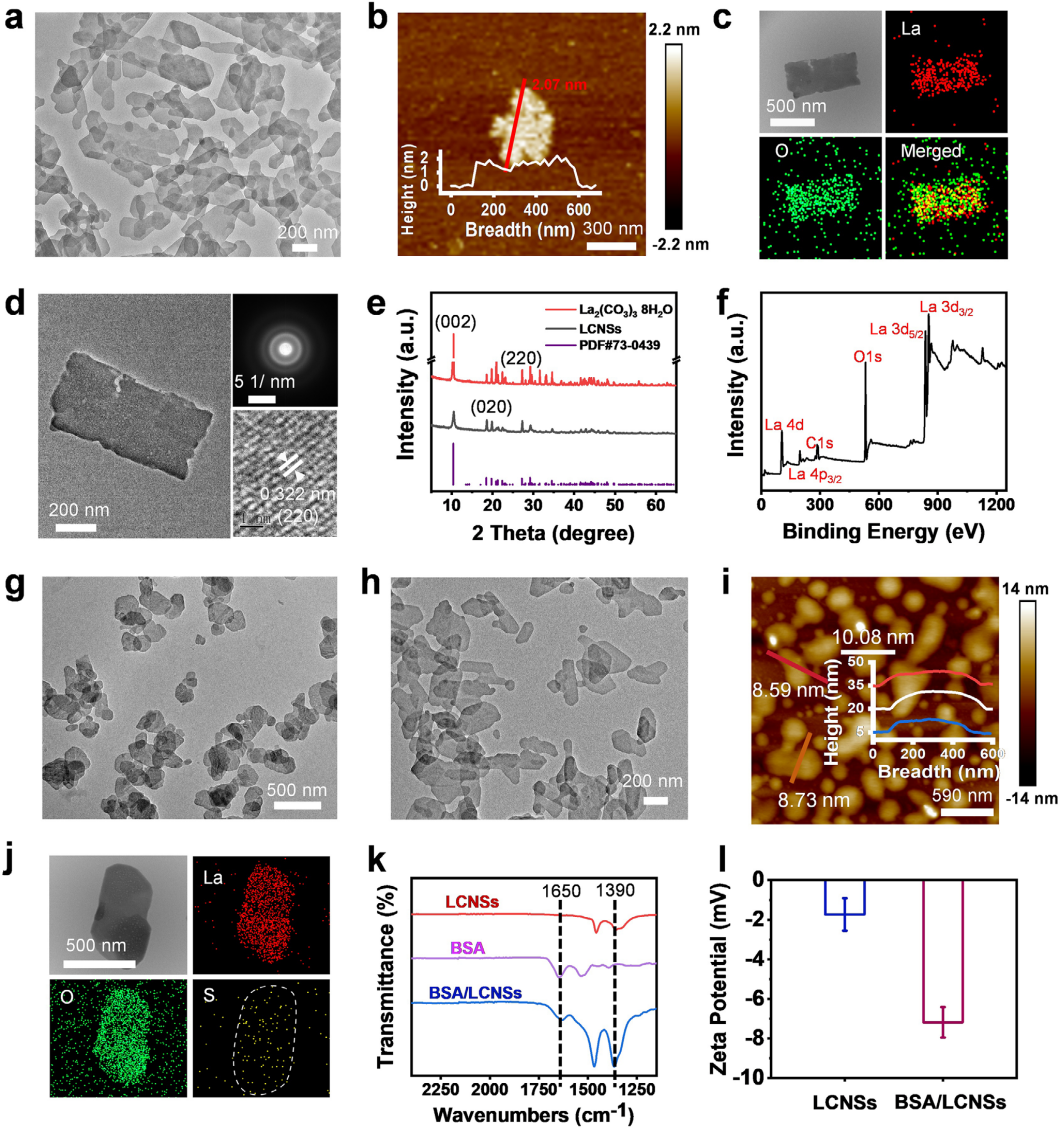
Characterization of BSA/LCNSs. (a) TEM image, (b) AFM image, (c) element mapping, and (d) HRTEM image of LCNSs. (e) X‐ray diffraction pattern (XRD) of LCNSs and La_2_(CO_3_)_3_·8H_2_O. (f) XPS spectra of LCNSs. (g) TEM images of LCNSs that were prepared without the addition of PVP and (h) with PVP. (i) AFM image of BSA/LCNSs, and (j) elemental mapping. (k) FTIR spectra analysis of BSA, LCNSs, and BSA/LCNSs. (l) Zeta potentials of LCNSs and BSA/LCNSs (*n* = 3).

During the preparation of nanosheets, PVP was introduced as an exfoliating agent, which facilitated the formation of thin nanosheet layers [24], as shown in Figure 2g. Furthermore, the removal of PVP molecules was accomplished through centrifugal washing (Figure S3). To enhance the aqueous dispersity of LCNSs and fabricate colloidal hydrogels with superior homogeneity, bovine serum albumin (BSA) was conjugated onto the surface of LCNSs. TEM and Scanning electron microscopy (SEM) analysis demonstrated that the BSA/LCNSs retained a sheet structure largely consistent with LCNSs (Figure 2h and Figure S4), with a significant increase in nanosheet thickness (∼9 nm) ascribed to the coverage of BSA molecules (Figure 2i). EDS elemental mapping confirmed the uniform distributions of La and O elements in BSA/LCNSs. Additionally, the detection of sulfur (S) elements on the nanosheets was correlated with the disulfide bonds present in BSA (Figure 2j). Fourier Transform Infrared (FTIR) spectra showed that the peak at 1390 cm^−1^ corresponded to the υ_3_ mode of the CO_3_^2−^ group, while the peak at 1650 cm^−1^ was identified as the amine peak in BSA (Figure 2k), indicating successful BSA modification which was then further supported by thermogravimetric (TG) analysis (Figure S5) and the zeta potential measurements (Figure 2l).

### 2.2 Preparation and Characterization GDLC Hydrogel

For the fabrication of colloidal GDLC hydrogel, homogeneous spherical Gela NPs were then prepared [22]. TEM image and size distribution analysis demonstrated that Gela NPs possess an average particle size of 279 nm (Figure 3a and Figure S6). Subsequently, Gela NPs, BSA/LCNSs (Figure 3b), and DAC were mixed under alkaline conditions. Modulating the solution pH could alter the interparticle interactions, resulting in the robust association of Gela NPs and BSA/LCNSs (Figure 3c), which subsequently crosslinked to form colloidal hydrogels (Figure 3d). As shown in Figure 3e, the zeta potential of BSA/LCNSs remained negative during the solution pH reduction process due to the presence of BSA (with an isoelectric point of 4.7), while the zeta potential of Gela NPs derived from gelatin type‐A (with an isoelectric point of about 9.0) reversed, causing the occurrence of nanoparticle attraction and aggregation (Figure 3f,g). The pH adjustment of the entire system was achieved through the gradual hydrolysis of D‐(+)‐glucitol‐lactone (GDL) in alkaline conditions, leading to the release of gluconic acid and a gradual decrease in solution pH, ultimately stabilizing around 7.0 (Figure 3h). The mechanical characteristics of GDLC hydrogels can be notably enhanced through the modulation of pH levels (Figure 3i and Figure S7) due to electrostatic interactions between the Gela NPs and BSA/LCNSs. Furthermore, the employment of GDL as a protein coagulant enhanced the cohesion of Gela NPs, resulting in superior elastic properties of the colloidal hydrogels (Figure S8). The GDLC hydrogel, incorporating LCNSs showed increased elastic modulus (G') and viscous modulus (G'') (Figure 3j), which may be attributed to the larger specific surface area of the nanosheets.

**FIGURE 3:**
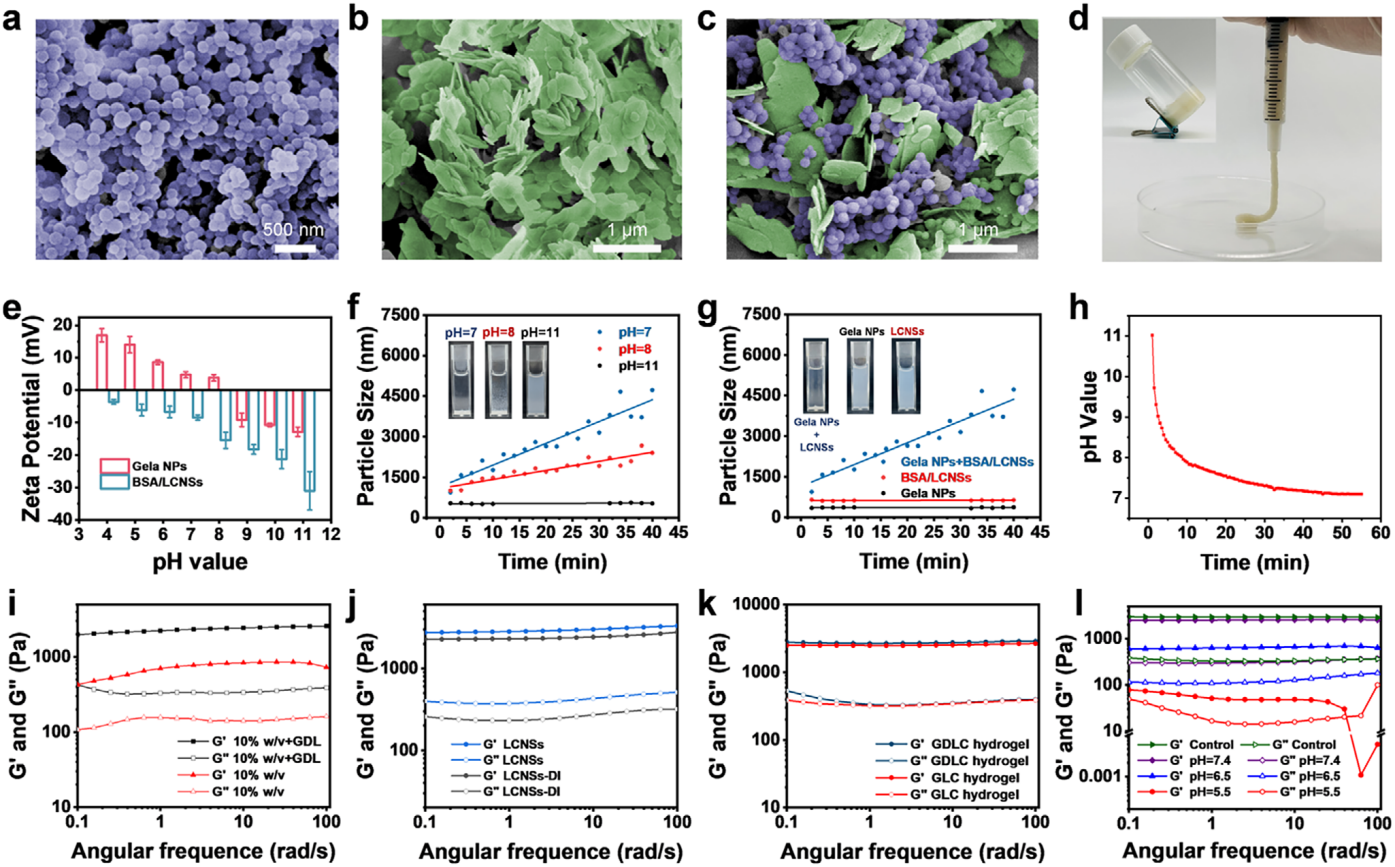
Characterization of GDLC hydrogel. SEM image of (a) Gela NPs, (b) BSA/LCNSs, and (c) GDLC hydrogel. (d) Photographs of GDLC hydrogel. (e) Zeta potential of Gela NPs and BSA/LCNSs at different pH levels (*n* = 3). (f) Particle sizes of Gela NPs mixed with BSA/LCNSs at pH = 7, pH = 8, and pH = 11. (g) Particle sizes of Gela NPs, BSA/LCNSs, and Gela NPs mixed with BSA/LCNSs at pH = 7. (h) The dynamic pH value of the system after adding GDL. (i) The rheological properties of 10% GDLC hydrogel with and without GDL. (j) Rheology of colloidal hydrogels prepared using LCNSs and LCNSs‐DI (LCNSs prepared in DI water). (k) Rheology of hydrogels loaded with/without DAC molecules. (l) Rheology after different pH treatments of GDLC hydrogel.

The viscoelastic characteristics of GDLC hydrogel were analyzed through rheological evaluations to determine the influence of solid content and formulation. The G' and G'' of the hydrogel progressively increased with rising solid content and remained stable (Figure S9). A reduction in the proportion of Gela NPs while maintaining a constant concentration resulted in a gradual decrease in G' and G'' intensity, leading to diminished stability (Figure S10). Thus, the optimal composition for GDLC hydrogel is identified as a 1:1 mass ratio of LCNSs to Gela NPs, with a preference for a higher prevalence of LCNS as the La^3+^ carrier within the hydrogel. In addition, the mechanical strength of the hydrogel was minimally impacted after loading with DAC (Figure 3k and Figure S11). Post‐treatment of the GDLC hydrogel under acidic pH conditions led to a significant reduction in its G' and G'' as well as stability, indicating the susceptibility of colloid hydrogel to acidic environments (Figure 3l and Figure S12). Additionally, the GDLC hydrogel demonstrated self‐healing capabilities (S13). In summary, GDLC hydrogel is anticipated to find potential applications in biomedical fields due to its superior mechanical properties, acid responsiveness, and self‐healing characteristics.

### 2.3 DAC/La^3+^ Release Profiles and Biocompatibility of GDLC Hydrogel

The TME has been demonstrated with elevated levels of MMP‐2/9 and lactic acid [25, 26]. MMP‐2/9, categorized as collagenase IV (also known as gelatinase), facilitated the degradation of Gela NPs under in vitro conditions (Figure 4a and Figure S14). Collagenase IV enzymatically disrupted the crosslinked network of colloidal hydrogel, resulting in an increased release of DAC (Figure 4b). Additionally, LCNSs within GDLC hydrogels underwent degradation and network disruption under acidic conditions, enhancing DAC release, with a release profile that exhibited pH dependency (Figure 4c). The acidic degradation of LCNSs led to La^3+^ release, also pH‐dependent, with up to 90% released within one day at pH 5.5 (Figure 4d). In normal mice, La^3+^ release from the colloidal hydrogel was minimal, whereas in the acidic TME of tumor‐bearing mice, La^3+^ showed a sustained release of approximately 60% over 17 days (Figure 4e). GDLC hydrogels demonstrated degradability in the tumor microenvironment, significantly reducing in size near the tumor until complete degradation (Figure 4f). By day 14, the GDLC hydrogel was entirely degraded, though more La^3+^ remained at the injection sites, likely due to its accumulation in tumor tissue [27].

**FIGURE 4:**
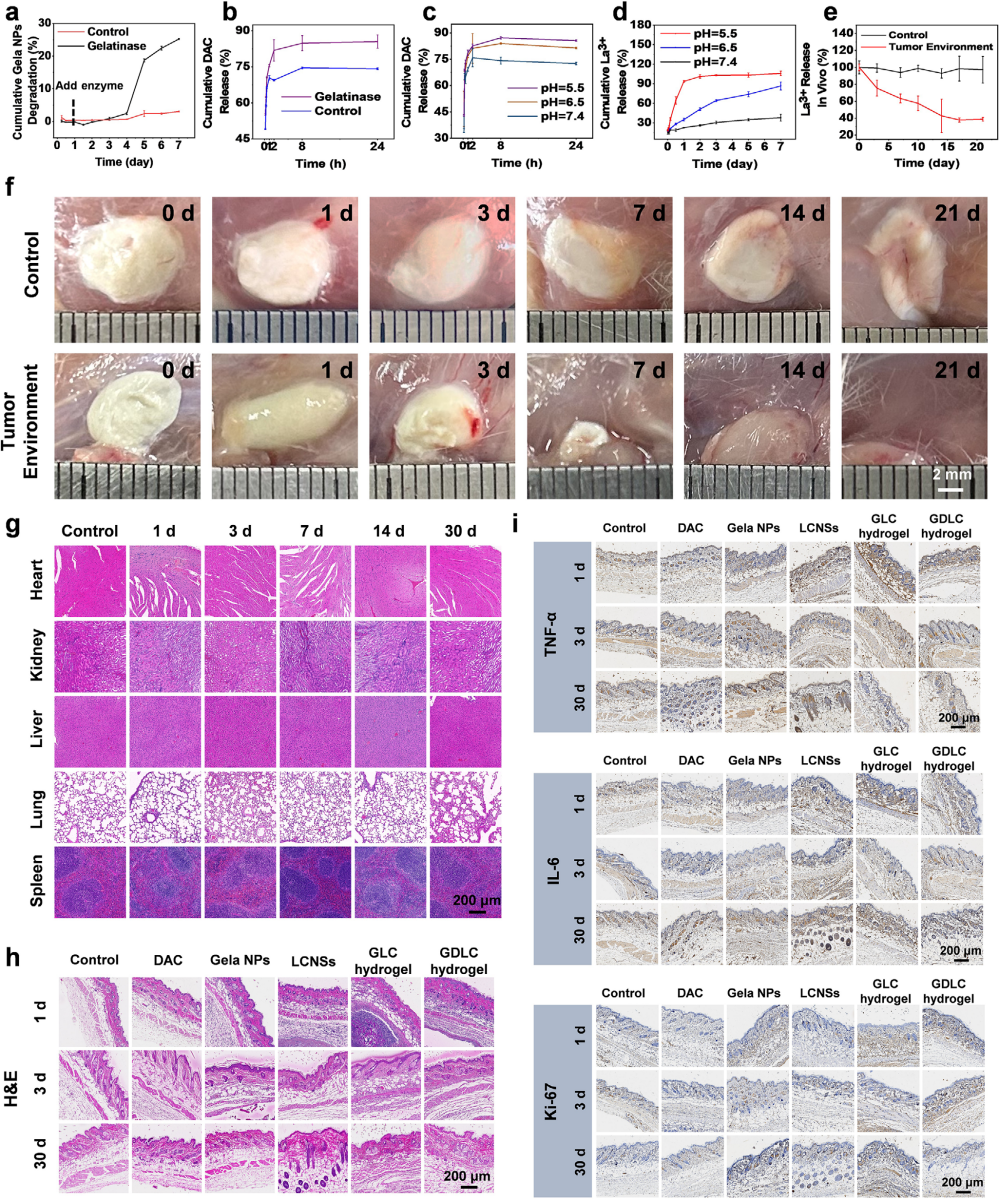
Drug release and biocompatibility. (a) Degradation of Gela NPs with and without gelatinase (*n* = 3). (b) DAC release from GDLC hydrogel with and without gelatinase (*n* = 3). (c) DAC and (d) La^3+^ release from GDLC hydrogel at different pH levels (5.5, 6.5, and 7.4) (*n* = 3). (e) In vivo La^3+^ release from GDLC hydrogel in tumor environment (*n* = 3). (f) Photographs of GDLC hydrogel degradation in vivo at different time points. (Scale bar: 2 mm). (g) H&E staining of the heart, liver, spleen, lung, and kidney of mice at various time points following GDLC hydrogel treatment. (h) H&E staining of skin samples from mice treated with different injections. (i) Immunohistochemical analysis of IL‐6, TNF‐α, and Ki‐67 in the injected skin of mice treated with different agents.

As a potential implant for in vivo application, a comprehensive biosafety evaluation must be conducted prior to confirming its anticancer efficacy. Preliminary safety assessments were performed in both normal mice and 4T1 tumor‐bearing mice. The results showed that, compared with the control group, no significant differences were observed in any of the hematological or blood biochemical parameters in the GDLC hydrogel‐treated group, indicating that no obvious hepatorenal toxicity or systemic damage was caused. To further assess the risk of off‐target drug accumulation in vital organs, the livers and kidneys of tumor‐bearing mice were collected on day 14, and the residual La^3+^ content was measured by inductively coupled plasma mass spectrometry (ICP‐MS). The La^3+^ content in the liver and kidney of the GDLC hydrogel‐treated group remained largely unchanged compared with that of the control group, showing no statistically significant difference. This indicated that, although the hydrogel was degraded and releases La^3+^ in the acidic tumor microenvironment, its accumulation in the liver and kidney is minimal and does not cause significant hepatotoxicity or nephrotoxicity (Figure S15). Histopathological examination revealed no lesions in vital organs over a period of 30 days (Figure 4g). To ascertain whether subcutaneous injection of GDLC hydrogel precipitated local dermal injury or inflammatory responses, skin tissues from the injection sites were collected at various time points for hematoxylin and eosin (H&E) staining and immunohistochemical analysis (Figure 4h,i). Comparative analysis of skin tissues between experimental groups and the control group within 1–30 days revealed no significant morphological alterations in the epidermis, dermis, and subcutaneous layers, with no detectable lesions. Immunohistochemical markers (IL‐6, TNF‐α, and Ki‐67) confirmed that the hydrogel did not elicit local inflammation or malignant cell proliferation. These findings collectively substantiated that GDLC hydrogel could serve as a promising biocompatible implant for controlled drug release within the TME.

### 2.4 Induction of 4T1 Cell Pyroptosis From GDLC Hydrogel

The anticancer mechanism of GDLC hydrogel was explored through an assessment of its cytotoxic effect on malignant cells to highlight its potential as an antineoplastic agent. At physiological pH 7.4, GDLC hydrogel demonstrated minimal cytotoxicity toward cancer cells, attributed mainly to the reduced release of La^3+^ compared to the pronounced cytotoxicity observed at acidic pH 6.5 (Figure 5a). Gela NPs and DAC exhibited no cytotoxic effects on cancer cells in either condition (Figure 5b, Figures S16 and S17). The toxicity of the GDLC hydrogel toward normal cells was further evaluated in this study. The experimental results showed that under pH 6.5 conditions simulating the acidic tumor microenvironment, the GDLC hydrogel had a certain effect on the viability of AML12 and RAW264.7 cells. Notably, under pH 7.4 conditions, the GDLC hydrogel exhibited only a very mild inhibitory effect on these two normal cell types. This difference fully demonstrated the acid‐responsive property of the GDLC hydrogel. These results indicated that the GDLC hydrogel had low cytotoxicity toward normal cells and possessed the potential for selective killing of tumor cells (Figure S18). The cell morphology in each treated group was analyzed using STYOX localization. Under both pH conditions (pH 7.4 and 6.5), cells treated with GDLC hydrogel displayed classical pyroptotic morphology, characterized by cell lysis and vesicle formation, whereas other groups did not exhibit any pyroptotic features (Figure 5c and Figure S19). Pyroptosis is characterized by the creation of membrane pores and the subsequent release of intracellular components, which is indicated by increased levels of LDH and ATP (Figure 5d,e). The GLC group (without DAC) displayed cell apoptotic characteristics such as intact membranes, chromatin condensation, and plasma membrane blebbing, as observed by bioelectron microscopy. In contrast, the GDLC hydrogel group exhibited distinct pyroptosis markers, including mitochondrial swelling and ruptured cellular membranes (Figure 5f) [28].

**FIGURE 5:**
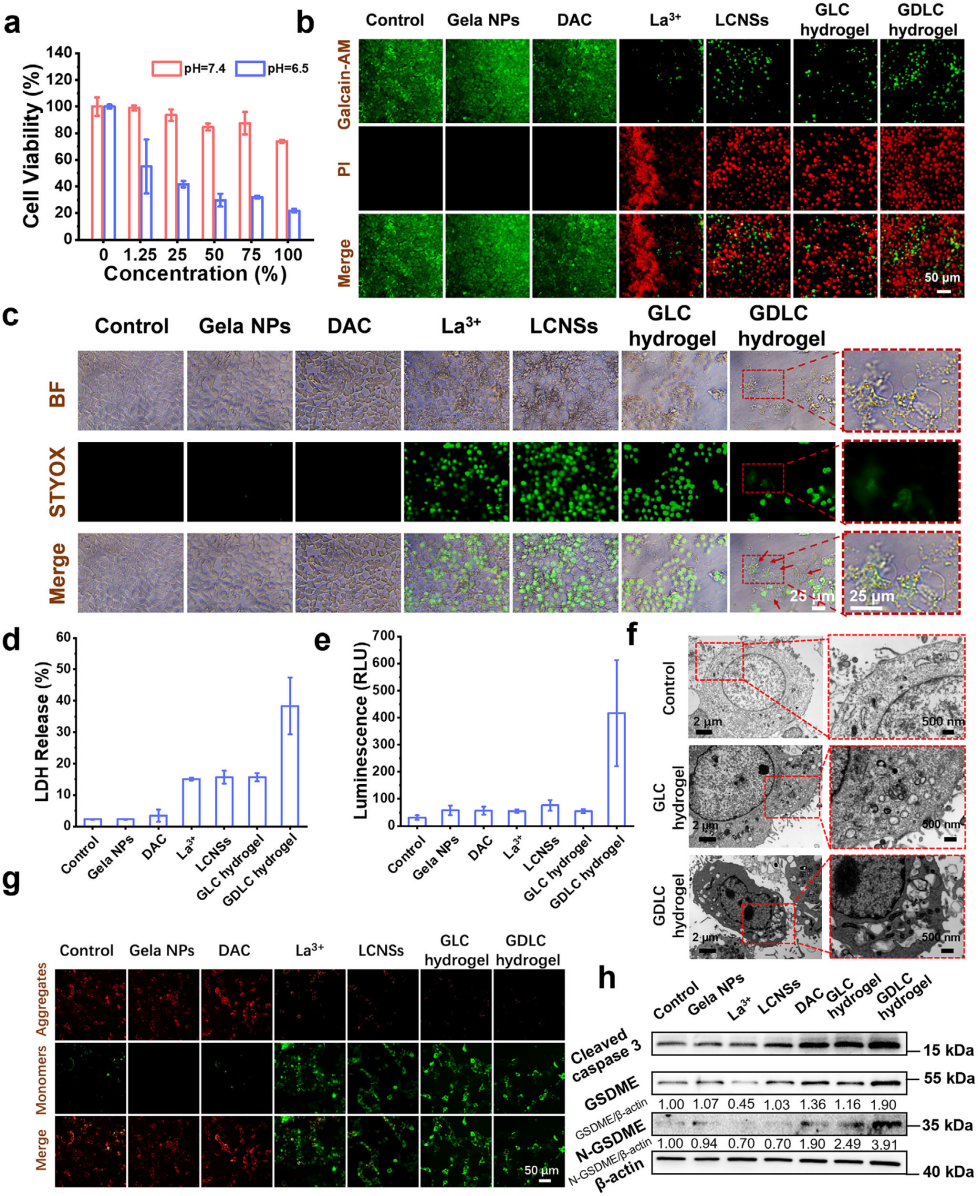
GDLC hydrogel induced 4T1 cell pyroptosis. (a) Cytotoxicity of GDLC hydrogel extracts at different pH values (6.5 and 7.4) for 4T1 cells (*n* = 4). (b) Galcain‐AM/PI (Calcein‐AM: live cells, PI: dead cells) staining of 4T1 cells after different treatments under mildly acidic conditions (pH = 6.5). (c) STYOX staining of 4T1 cells after various treatments under mildly acidic conditions (pH = 6.5). The red arrows indicated cells with damaged membranes. (d) LDH and (e) ATP release in 4T1 cells after different treatments under mildly acidic conditions (pH = 6.5) (*n* = 3). (f) BioTEM of 4T1 cells after various treatments. (g) JC‐1 staining of cells with various treatments. (h) Western blot analysis of pyroptosis.

Numerous investigations have demonstrated a strong association between GSDME‐mediated pyroptosis and the mitochondrial pathway [29]. To elucidate the influence of La^3+^ on mitochondrial function, the mitochondrial membrane potential was initially measured utilizing JC‐1 dye. La^3+^ compromised the integrity of mitochondrial membranes, as indicated by the prevalence of green fluorescence (Figure 5g). Western blot analysis of cells under different treatments showed that compared with the PBS control group, the GDLC hydrogel containing La^3^^+^ and DAC increased the band intensity of GSDME by 1.9‐fold and that of N‐GSDME by 3.91‐fold, suggesting activation of the GSDME‐caspase‐3‐mediated pyroptosis pathway (Figure 5h). To preliminarily verify the immunomodulatory effects of the GDLC hydrogel, in vitro experiments were performed using mouse bone marrow‐derived dendritic cells (BMDCs) and RAW264.7 macrophages. The GDLC hydrogel was soaked in culture medium at pH 6.5 for 24 h, and the extract was collected. BMDCs and RAW264.7 cells were then treated with the extract for 24 h. Flow cytometry was used to detect the expression of CD80/CD86 on BMDCs and M1/M2 markers on RAW264.7 cells. In the GDLC treatment group, the proportion of CD80^+^CD86^+^ double‐positive cells was 5.45 times that of the control group, indicating that DAC and La^3+^ released from GDLC synergistically promoted DC maturation. Additionally, the proportion of M1‐type macrophages in the GDLC treatment group was significantly increased, and the M1/M2 ratio was approximately 7.56 times higher than that of the control group (Figure S20). These in vitro results directly demonstrated that the GDLC hydrogel can significantly promote the maturation of BMDCs and the M1 polarization of macrophages.

### 2.5 RNA Sequencing

To further delineate the therapeutic mechanism of GDLC hydrogel in breast carcinoma cells, this study employed transcriptome profiling to discern mRNA variants in 4T1 cancer cells subjected to GDLC hydrogel treatment. After unsupervised hierarchical clustering, the distinct aggregation of data from identical treatment groups underscores the high reliability of the RNA sequencing data (Figure S21). A comprehensive analysis revealed the identification of 12 592 genes, among which 1971 were found to be differentially expressed when comparing the GDLC hydrogel‐treated group to the control group. Among them, 476 genes (24.1%) were upregulated, while 1,495 genes (75.9%) were downregulated (Figure 6a,b and Figure S22). Subsequently, Kyoto Encyclopedia of Genes and Genomes (KEGG) pathway analysis was conducted (Figure 6c), revealing that Differentially Expressed Genes (DEGs) associated with GDLC hydrogel treatment were predominantly enriched in the Toll‐like receptor pathway, NF‐κB pathway, interleukin‐17 (IL‐17) pathway, and NOD‐like receptor pathway, suggesting a fundamental linkage between GDLC hydrogel intervention and inflammation (Figure S22). Cellular pyroptosis, a mechanism intricately linked to inflammation, is pivotal for initiating immune responses [30]. Gene Ontology (GO) analysis (Figure 6d) of GDLC hydrogel‐treated cells corroborated that these DEGs are implicated in inflammation‐related pathways and immune responses. The ring numeric heatmap (Figure 6e) demonstrated upregulated signals, encompassing inflammation‐associated genes (Srsf1, Card6, Tgfb3, Il1r1, Pparg) and immune‐related genes (Il4, Hspa1b, Hspa8, Cd24a) [31]. Additionally, the protein‐protein interaction network map highlighted interaction involving inflammation and immune response‐related proteins (Figure 6f), indicating that GDLC hydrogel treatment augments inflammatory and immune‐related interactions.

**FIGURE 6:**
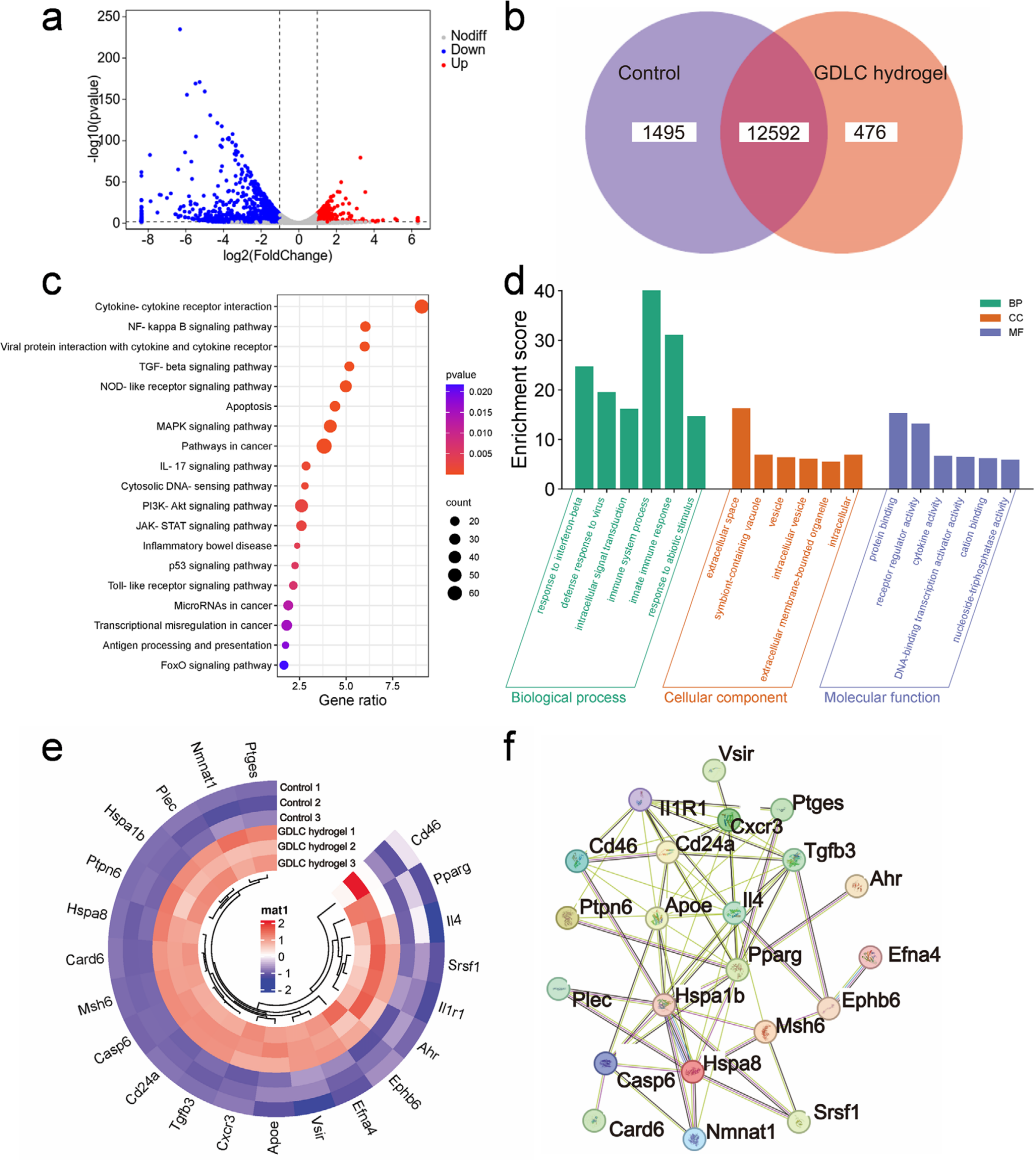
RNA sequencing of 4T1 cells after different treatments. (a) Volcano map comparing the GDLC hydrogel group to the control group. (b) Venn diagram expressed in Control and GDLC hydrogel groups. (c) KEGG and (d) GO enrichment analysis after GDLC hydrogel treatment. (e) A ring numeric heatmap analysis of genes involved in inflammation and immune. (f) Protein‐protein interaction network of these functional genes.

### 2.6 Evaluation of Anti‐Tumor Effect of GDLC Hydrogel

The aforementioned studies preliminarily indicated that the GDLC hydrogel, functioning as an acid‐responsive agent, could induce pyroptosis and hold promise for in vivo antitumor treatment. To substantiate its anticancer efficacy, GDLC hydrogel and its constituent fractions were administered peritumorally to subcutaneous tumors in a murine mammary carcinoma model, following the specified protocol (Figure 7a). Tumor bioluminescence imaging (Figure 7b), tumor volume measurements (Figure 7c), and survival rates of the mice (Figure 7d) revealed a marked enhancement in tumor suppression and survival in GDLC hydrogel‐treated 4T1 tumor‐bearing mice compared to the control group. The superior therapeutic outcomes observed in the La^3+^ + DAC and GDLC hydrogel groups underscore the pivotal role of pyroptosis in cancer therapy. As the expression of inhibitory immune checkpoints significantly influences the efficacy of cancer immunotherapy [32], the combination treatment of GDLC hydrogel and aTIGIT (GDLC hydrogel + aTIGIT) exhibited heightened therapeutic efficacy, manifested by reduced tumor volumes and more pronounced tumor growth inhibition (Figure 7e and Figure S23). Treated mice demonstrated significantly elevated levels of inflammatory factors (Figure 7f). Immunohistochemical analyses of tumors from different treatment groups, including Ki‐67, HMGB1, caspase‐3, and GSDME, along with immunofluorescence staining of CD8 and CD49b molecules (Figure 7g), corroborated the in vitro findings of caspase‐3 and GSDME, indicating localized cellular pyroptosis during treatment. GDLC hydrogel‐induced pyroptosis upregulated and extracellularly releases HMGB1. Ki‐67 results mirrored the tumor growth inhibition findings. Tumors from the GDLC hydrogel, aTIGIT, and GDLC hydrogel + aTIGIT groups showed increased fluorescence intensity of CD8 (red) and CD49b (green), signifying enhanced infiltration of T cells and Natural Killer cells (NK cells). Consequently, the initiation of cellular pyroptosis and the subsequent release of inflammatory mediators enhance tumor immunogenicity, thereby promoting the recruitment and activation of immune cells.

**FIGURE 7:**
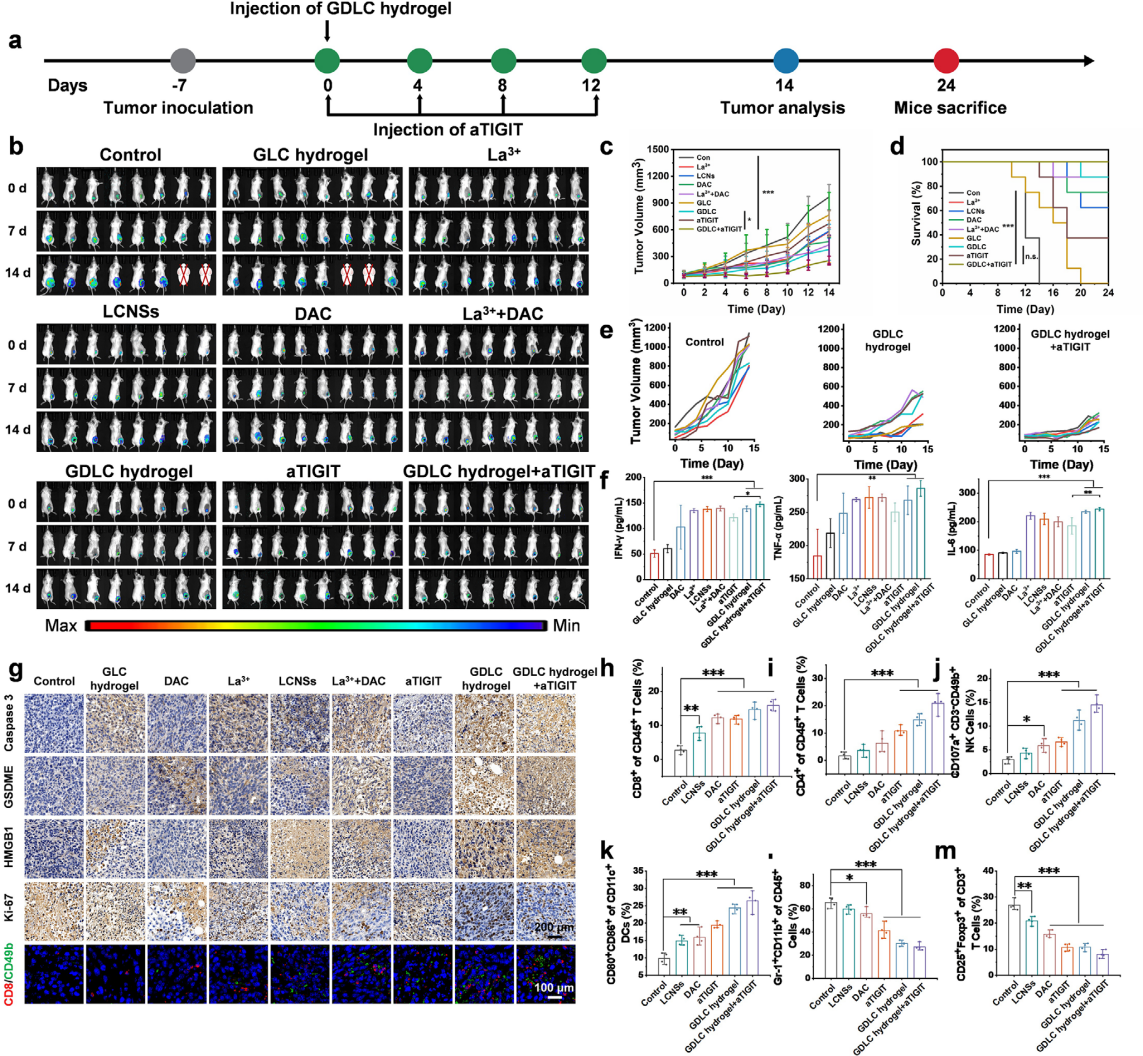
Anti‐tumor effect of GDLC hydrogel. (a) Schematic design of GDLC hydrogel‐induced cellular pyroptosis for anti‐tumor therapy experiment. (b) Representative bioluminescence images of mice in each treatment group taken on days 0, 7, and 14. (c) Mean tumor volume curves, (d) survival rates, and (e) individual tumor growth curves of 4T1‐tumor‐bearing mice in different groups (*n* = 8). (f) Serum inflammatory factors (IFN‐γ, TNF‐α, and IL‐6) were measured following various treatments (*n* = 3). (g) Immunohistochemical analysis (Ki‐67, HMGB1, caspase‐3, and GSDME) and immunofluorescence analysis (CD8 and CD49b) of tumors in different treatment groups. Flow cytometric analysis of tumor‐infiltrated immune cells (h) CD4^+^ T cells, (i) CD8^+^ T cells, (j) NK cells, (k) DCs, (l) MDSCs, and (m) Tregs in different treatment groups. ^*^ *p* < 0.05, ^**^ *p* < 0.01, and ^***^ *p* < 0.001.

Immunological activating mechanism of the GDLC hydrogel was further evaluated in murine models bearing breast carcinoma post‐treatment. Treatments regimens involving GDLC hydrogel, aTIGIT, and the combination of GDLC hydrogel + aTIGIT resulted in a marked augmentation in the infiltration of CD8^+^ and CD4^+^ T cells within the tumor microenvironment, with these increases being several‐fold higher in comparison to the control group (Figure 7h,i and Figure S24a). Additionally, there was a notable enhancement in the infiltration of NK cells as observed in the GDLC hydrogel + aTIGIT group (Figure 7j and Figure S24b). The GDLC hydrogel group demonstrated a mature Dendritic cells (DCs) proportion as high as 25%, indicating the initiation of an adaptive immune response and improved antigen presentation (Figure 7k and Figure S24c). In contrary, regulatory T cells (Tregs) and myeloid‐derived suppressor cells (MDSC), known for their immunosuppressive role in oncological immunotherapy [33], were significantly reduced in the GDLC hydrogel and GDLC hydrogel + aTIGIT groups (Figure 7l,m and Figure S24d,e). These findings indicated that GDLC hydrogel may enhance cellular immune responses against tumors by inducing cellular pyroptosis, thereby demonstrating considerable therapeutic potential in the field of cancer immunotherapy.

### 2.7 Inhibition of Distant Tumor

During oncological treatment, the emergence of metastasis would lead to therapeutic failure [34]. To investigate the efficacy of various treatments on metastatic tumors, a dual‐tumor model (with approximately 1 × 10^6^ cells injected at each end) was established to simulate the spread of cancer. Various agents were administered peritumorally to the primary tumors, and their inhibitory effects on the growth of distant tumors were also monitored (Figure 8a). Tumor progression was markedly inhibited in the groups receiving GDLC hydrogel + aTIGIT treatment after 14 days. Notably, the dimensions of primary tumors in both experimental groups were marginally smaller than that of distant tumors, potentially attributable to the direct induction of apoptosis or pyroptosis (Figure 8b–d and Figure S25). The survival rates of murine subjects mirrored the observed tumor growth suppression (Figure 8e). To elucidate the underlying mechanisms of the therapeutic efficacy against distant tumors within the dual‐tumor model, flow cytometric analysis revealed a significant augmentation of CD8^+^ and CD4^+^ T cell infiltration in both primary and distant tumors, demonstrating a substantial increase of immuno‐stimulating T cells in comparison to the control group (Figures S26 and S27). NK cell infiltration was also significantly increased in all treated groups compared to the control group, with a higher infiltration observed in primary tumors. Additionally, the maturation of DCs in both tumors exhibited a remarkable increase in the GDLC hydrogel + aTIGIT group compared to the control group. In both primary and distant tumors, there was a comparable decline in the levels of Tregs and MDSC, with their quantities being significantly diminished in comparison to the control group. The decrease in immunosuppressive cells within distant tumors could potentiate the anti‐tumor immune response, culminating in an enhanced therapeutic outcome (Figure 8f,g and Figure S27).

**FIGURE 8:**
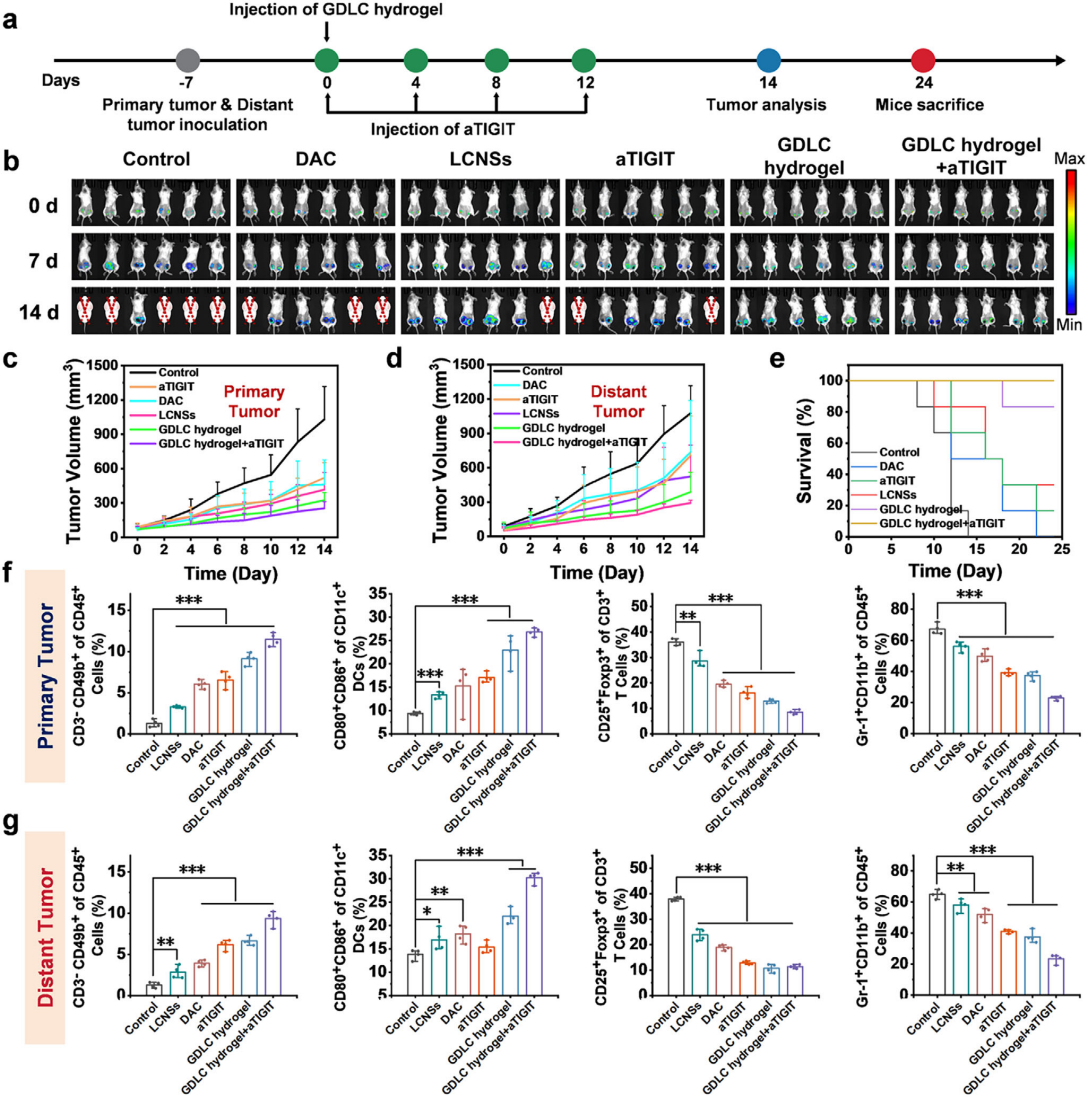
Inhibition of distant tumors. (a) Schematic illustration of the experimental design for evaluating the inhibitory effect of GDLC hydrogel on distant tumors in a bilateral 4T1 tumor‐bearing model. (b) Representative bioluminescence images of mice in each treatment group taken on days 0, 7 and 14. (c) Mean tumor volume curves of primary tumors over time for each group, (d) Mean tumor volume curves of distant tumors over time for each group, and (e) survival rates of 4T1‐tumor‐bearing mice for each group (*n* = 6). Flow cytometric analysis of NK cells, DCs, MDSCs, and Tregs in (f) primary tumors and (g) distant tumors from different treatment groups (*n* = 4). ^*^ *p* < 0.05, ^**^ *p* < 0.01, and ^***^ *p* < 0.001.

### 2.8 Inhibition of Tumor Recurrence and Metastasis After Surgery

Surgical intervention for cancer presents considerable therapeutic benefits, but residual local tumor tissues and circulating tumor cells during the procedure still can lead to post‐resection recurrence [35]. The efficacy of GDLC hydrogel as a pyroptosis‐inducing agent in mitigating postoperative tumor recurrence and metastasis was evaluated using a postoperative tumor model. The individual components were applied to the surgical sites to assess their impact on tumor recurrence and metastasis (Figure 9a). GDLC hydrogel induced pyroptosis in residual tumor cells, eliciting a potent anti‐tumor immune response against remaining tumor cells, which was further amplified by the addition of aTIGIT. In the GDLC hydrogel group, two mice, and in the GDLC hydrogel + aTIGIT treatment group, five mice displayed no tumor recurrence (n = 8) (Figure 9b,c). Furthermore, recurrent tumors in both the GDLC hydrogel and GDLC hydrogel + aTIGIT groups exhibited reduced volumes and extended survival rates compared to the control group (Figure 9d,e). Histopathological examination of lung tissues revealed an absence of metastatic nodules in the GDLC hydrogel + aTIGIT group, while smaller metastatic nodules were observed in the GDLC hydrogel group, and the control group presented with extensive metastatic nodules. Additionally, there was a significantly increased infiltration of CD8^+^ T cells (red) and CD49b^+^ NK cells (green) in the aTIGIT, GDLC hydrogel, and GDLC hydrogel + aTIGIT groups, indicating the enhancement of anti‐tumor immunity (Figure 9f). In summary, GDLC hydrogel effectively could induce pyroptosis in vivo, augment the immune response when combined with aTIGIT, and thereby suppress tumor proliferation and pulmonary metastasis.

**FIGURE 9:**
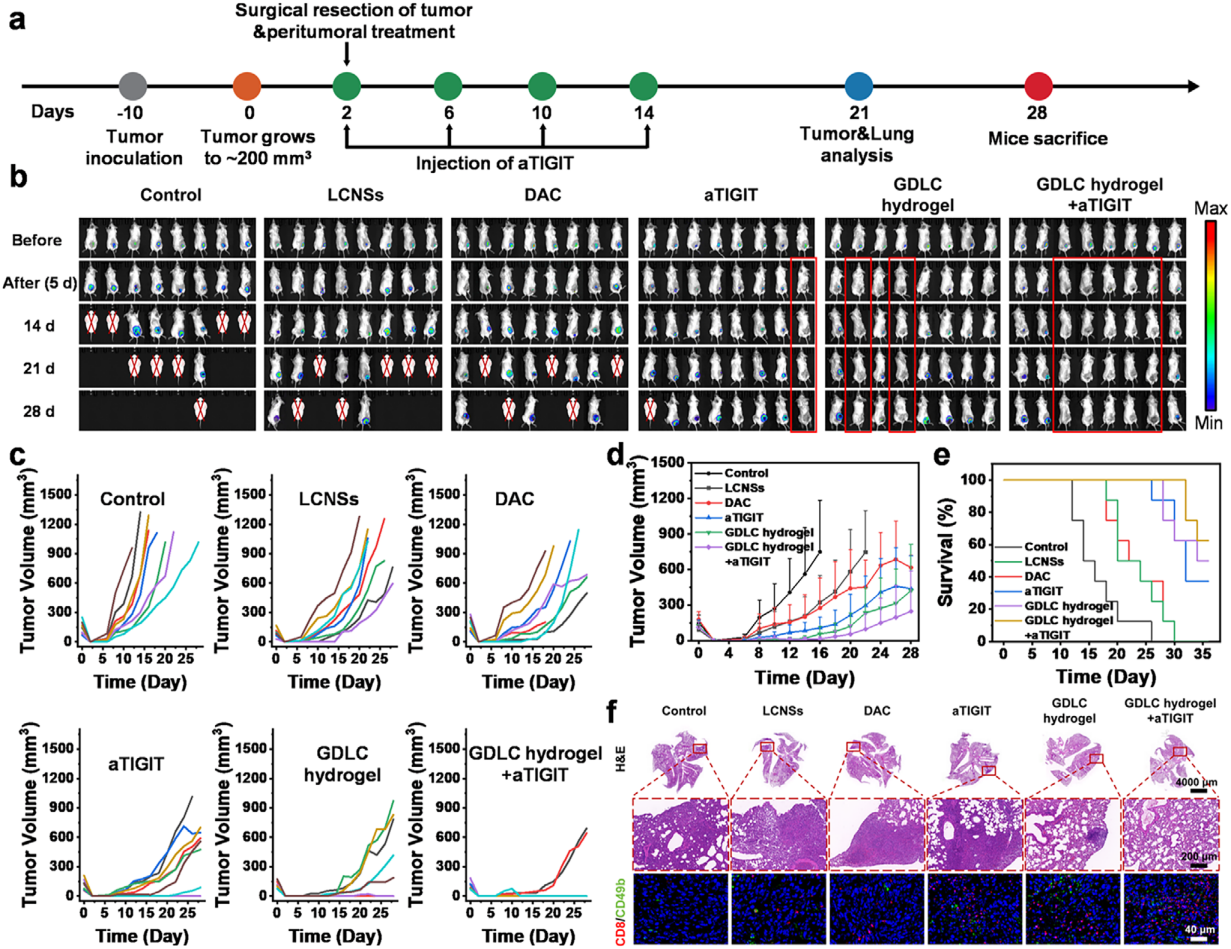
Prevention of postoperative tumor recurrence and metastasis by GDLC hydrogel. (a) Schematic representation of the experimental design for the postoperative application of GDLC hydrogel. (b) Representative bioluminescence images of mice in each treatment group taken on days 0, 5, 14, 21 and 28. (c) individual tumor growth curves. (d) Mean tumor volume curves, and (e) survival rates of post‐operative mice in different groups (*n* = 8). (f) Lung H&E staining and tumor immunofluorescence staining of tumor sections for CD8 (red) and CD49b (green) to visualize CD8^+^ T cells and NK cells, respectively.

### 2.9 Long‐Term Immune Memory Effect

To ascertain the sustained anti‐tumor immunological memory effect, mice that had been cured using GDLC hydrogel combined with aTIGIT and surgical intervention were subjected to a secondary challenge of 4T1‐Luc cells, and tumor progression was meticulously tracked (Figure 10a and Figure S28). The treatment group demonstrated a statistically significant reduction in tumor recurrence and a decrease in tumor sizes when compared to the control group (Figure 10b). Moreover, the tumors that recurred in the treated mice exhibited significantly reduced growth rates (Figure 10c,d). Survival rates were notably improved in the group treated with GDLC hydrogel and aTIGIT molecules (Figure 10e). Flow cytometry of the lymph nodes proximal to the tumor site showed a substantial increase in both central memory T cells (T_CM_, CD44^+^CD62L^+^) and effector memory T cells (T_EM_, CD44^+^CD62L^−^) compared to the control group (Figure 10f, g and Figure S29). Additionally, T_EM_ levels in the spleen were significantly elevated compared to the control group (Figure 10h, i and Figure S29). The synergy of immunogenic pyroptosis and aTIGIT effectively stimulated robust anti‐tumor immunological memory, thereby mitigating the recurrence and metastasis of breast cancer in murine models.

**FIGURE 10:**
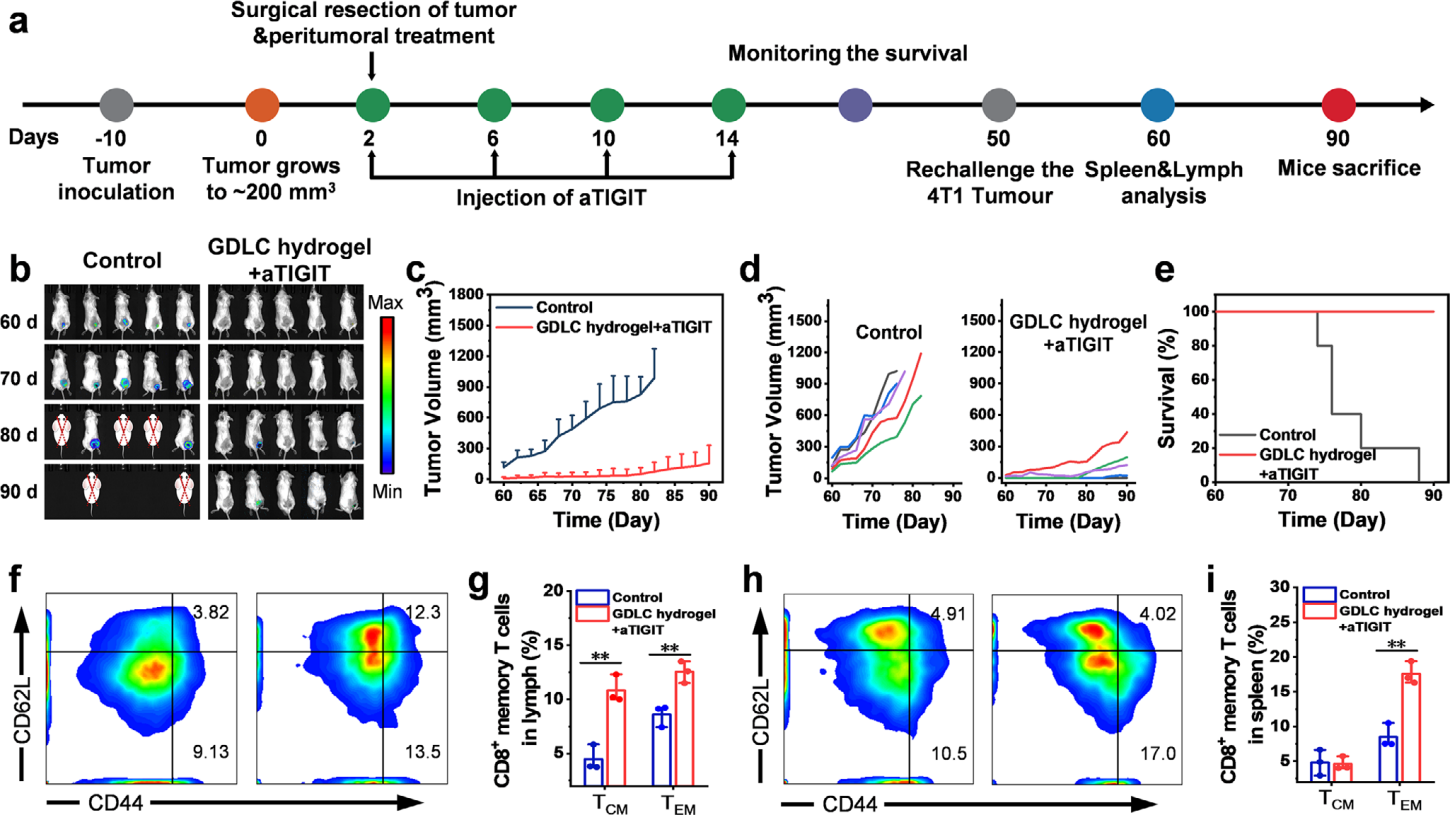
GDLC hydrogel + aTIGIT induced long‐term immune memory effect. (a) A schematic representation of the experimental design for the investigation of long‐term immune memory effects. (b) Representative bioluminescence images of mice in each treatment group taken on days 60, 70, 80, and 90. (c) Mean tumor volume curves and (d) individual tumor growth curves. (e) Survival rates of rechallenged mice in the control and GDLC hydrogel + aTIGIT group (*n* = 6). Flow cytometric analysis of T_CM_ and T_EM_ in (f,g) lymph nodes and (h, i) spleen (*n* = 3). ^*^ *p* < 0.05, ^**^ *p* < 0.01, and ^***^ *p* < 0.001.

## 3 Discussion and Conclusions

Immunotherapy constitutes a fundamental element in the therapeutic arsenal against various malignancies [36]. Immune checkpoint blockade (ICB) therapy, a promising modality in oncological treatment, functions by inhibiting the suppressive receptors on cytotoxic T‐lymphocytes (CTLs) [37]. The limited efficacy of T cell‐centered ICB in solid tumor management is largely attributed to the dependency of adaptive immunity on the antigen‐presenting capabilities of the innate immune system. Our previous study have identified a novel immune checkpoint inhibitor known as aTIGIT, which has shown substantial promise in oncological immunotherapy [38]. The identification of TIGIT on both T cells and NK cells, and the subsequent application of aTIGIT, was found to mitigate the immunosuppressive environment affecting both cell types, thereby facilitating a robust multi‐level anti‐tumor response through activating both innate and adaptive immunity. In this research, the combinatory therapeutic strategy integrating pyroptosis with aTIGIT was observed to augment the migration of T cells and NK cells from the tumor site and neutralize the immunosuppressive conditions within the tumor microenvironment, culminating in enhanced cancer immunotherapy outcomes.

Pyroptosis is a type of programmed cell death characterized by its immunogenic and pro‐inflammatory properties, which sets it apart from apoptosis [39]. Within the field of cancer immunotherapy research, the phenomenon of cellular pyroptosis has recently gained significant attention due to its potential to enhance therapeutic efficacy [40]. The epigenetic silencing of particular genes impedes the caspase‐3/GSDME‐mediated activation of cellular pyroptosis in certain malignancies, although it generally augments apoptosis by promoting caspase‐3 activation. The role of DAC transcends the limitations inherent in strategies centered on cellular focalization within cancer immunotherapy for these specific cancer types. In this investigation, DAC administration effectively initiated caspase‐3/GSDME‐mediated pyroptosis in 4T1 cells, facilitating the shift from apoptosis to pyroptosis. These findings also suggest the prospective utility of GDLC hydrogel in other cancer immunotherapies where specific pyroptosis genes are epigenetically silenced. Notably, the induction of pyroptosis by GDLC hydrogel was not complete, attributable to the high efficacy of DAC. Nonetheless, the anti‐tumor immune response elicited by GDLC hydrogel‐induced pyroptosis was sufficiently robust to markedly suppress tumor proliferation, owing to the beneficial anti‐tumor immune feedback mechanism triggered by the release of inflammatory mediators during pyroptosis. These results align with recent studies indicating that pyroptosis in less than 15% of tumor cells is adequate to control the progression of 4T1 breast tumors [41]. This strategy of inducing limited tumor cell pyroptosis to activate anti‐tumor immunity is advantageous in clinical applications as it significantly mitigates treatment‐related toxic side effects, corroborated by the favorable biocompatibility outcomes of GDLC hydrogel observed in our study.

In this study, lanthanum carbonate acts as a carrier of La^3+^ ions and facilitates the formation of cross‐linked colloidal gel structures, endowing the hydrogels with multiple functionalities such as pH‐responsive drug delivery and controlled La^3+^ release. Employed in the engineering of colloidal hydrogel platforms, lanthanum carbonate exhibits significant potential as an optimal material for in vivo implantation and can be leveraged for cancer immunotherapy by inducing cellular pyroptosis. Despite these promising findings, the complete therapeutic mechanisms of lanthanide‐based ions in GDLC hydrogel for cancer treatment remain to be fully elucidated. For instance, La^3+^ has been shown to inhibit cancer cell invasion and migration by blocking the NF‐κB signaling pathway and down‐regulating the expression of MMP‐1 and MMP‐9 [42], thereby opening new avenues for advanced cancer treatment strategies utilizing GDLC hydrogel.

In conclusion, this study successfully developed a homogeneous colloidal hydrogel platform utilizing LCNSs and Gela NPs, which, when loaded with DAC, can mitigate the methylation of pyroptosis‐related genes in breast cancer cells, activate caspase‐3 via La^3+^ interaction, and demonstrate safety for application. The induction of cellular pyroptosis‐mediated immunogenic cell death and intrinsic adjuvant properties can counteract the tumor's immunosuppressive microenvironment and potentiate the adaptive anti‐tumor immune cascade response. This approach can synergize with immune checkpoint inhibitors to effectuate a robust anti‐tumor response and amplify immune activation in a dual‐tumor model of subcutaneous murine breast cancer. Furthermore, it efficiently promotes the differentiation of immune memory T cells, leading to a sustained immune memory effect that effectively prevent cancer recurrence. Thus, our study suggested that triggering pyroptosis in breast cancer cells through epigenetic mechanisms can result in accelerated response times and heightened immune efficacy, offering significant insights into tumor immunotherapy. Although the current study was based on a subcutaneous tumor model, the establishment of an orthotopic breast cancer model will be prioritized in future work to further validate the anti‐tumor efficacy, local pharmacokinetics, and immunomodulatory effects of the GDLC hydrogel in a more clinically relevant environment, thereby providing stronger evidence for clinical translation. These findings offer promising opportunities to enhance conventional cancer therapies and facilitate their translation into clinical practice.

## 4 Experimental Section

### 4.1 Materials

Lanthanum chloride heptahydrate, lanthanum acetate, guanidine hydrochloride, D‐(+)‐gluconic acid δ‐lactone, decitabine, and MTT were purchased from Aladdin (Shanghai, China). Sodium carbonate, acetone, glutaraldehyde, and DMSO were purchased from Sinopharm Chemical Reagent Co., Ltd (Shanghai, China). Gelatin type A was purchased from Sigma–Aldrich (USA). STYOX Green was purchased from ALPHABIO (Tianjin, China). Collagenase type IV was purchased from Biosharp (Beijing, China). Phosphate‐free RPMI 1640 medium was purchased from Coolaber (Beijing, China). TruStain FcX, 7AAD, APC‐CD8, APC‐Cy‐7‐CD4, FITC‐CD3, BV421‐CD44, PE‐CD62l, Alexa Fluor 700‐CD45, FITC‐CD11c, PE‐CD80, APC‐Cy7‐CD86, BV421‐CD11b, PE‐Foxp3, BV421‐CD11b, and APC‐Cy7‐Gr‐1 were purchased from BioLegend (USA). GSDME, caspase‐3, HMGB1, and ki‐67 were purchased from Proteintech (Wuhan, China). TNF‐α, IL‐6, and IFN‐γ ELISA Kits were purchased from Wuhan Colorful Gene Biotech Co., Ltd (Wuhan, China). D‐luciferin potassium salt was purchased from Macklin (China). aTIGIT was purchased from InVivoMAB (USA).

### 4.2 Synthesis of BSA/LCNSs and Gela NPs

2% solution of polyvinyl pyrrolidone (PVP) was prepared initially. Then, add 0.1 g of lanthanum acetate to 15 mL of PVP solution. In a separate container, add 0.05 g of sodium carbonate to 5 mL of PVP solution. Slowly add the sodium carbonate solution to the lanthanum acetate solution while using ultrasonication. Bovine serum albumin (BSA) was used to modify the LCNSs.

Gelatin nanoparticles (Gela NPs) were prepared using the acetone precipitation method. Under the conditions of a 40°C water bath, 1.8 g of type A gelatin was dissolved in 75 mL of deionized water with stirring. The pH was then adjusted to 2.3 using a diluted hydrochloric acid solution. Subsequently, 210 mL of acetone was slowly added dropwise, causing the solution to change from colorless and transparent to milky white. After cooling to room temperature, 495 µL of 25% glutaraldehyde solution was added for cross‐linking and fixation for 12 h. Subsequently, 50 mL of a 10% guanidine hydrochloride solution was added and stirred for 1 h. The Gela NPs precipitates were collected by centrifugation at 9000 rpm for 10 min and washed three times with deionized water.

### 4.3 Synthesis of GDLC Hydrogel

BSA/LCNSs and Gela NPs were separately dispersed in 0.5 mL of pH = 11 water. Subsequently, the BSA/LCNSs and Gela NPs were mixed together in a 1:1 volume ratio. 40 mg of DAC and 3 mg of milled D‐gluconate δ‐lactone were added and stirred to dissolve. The mixture was then acidified and flocculated for 1 h at room temperature. Subsequently, the two nanoparticles were crosslinked to form a hydrogel via electrostatic interactions.

### 4.4 Characterization of Gela NPs, BSA/LCNSs, and GDLC Hydrogel

In order to demonstrate the successful preparation of the nanoparticles, Gela NPs, BSA/LCNSs, and GDLC hydrogel were characterized by using XRD (PANalytical, Netherlands), AFM (Bruker, Germany), XPS (Thermo Scientific, USA), SEM (Hitachi, Japan), and TEM (Thermo Scientific, USA). The modification of BSA on LCNSs was characterized using an FTIR spectrometer (Thermo Scientific, USA) and a thermogravimetric analyzer (PerkinElmer, USA). The particle size distribution and zeta potential of the nanoparticles were characterized using a nanoparticle size and zeta potential analyzer (Malvern Panalytical, UK).

### 4.5 GDLC Hydrogel Rheological Properties Testing

The rheological properties of hydrogels were characterized using the Anton‐Paar MCR 302e rotational rheometer. Set the oscillation mode. The frequency range is 0.1−100 Hz. The values of the energy storage modulus (G′) and loss modulus (G″) were recorded.

### 4.6 In Vitro Degradation of Gela NPs

Gela NPs were divided into two groups and dispersed in a phosphate buffer solution (PBS) at pH = 7.4. Gelatinase group was treated with collagenase IV on day 1, while the control group was left untreated. 20 µL of supernatant was collected on days 0, 1, 2, 3, 4, 5, 6, and 7 for testing the free protein concentration to characterize gelatin nanoparticle degradation. Protein concentration was measured using a BCA protein concentration assay kit.

### 4.7 In Vitro DAC Release From GDLC Hydrogel

Initially, PBS was prepared at pH = 5.5, 6.5, and 7.4. The GDLC hydrogel was enclosed within dialysis bags characterized by a molecular weight cut‐off of 3000 and was subsequently fastened using clips. Subsequently, the bags were submerged in PBS at different pH levels and placed on a shaker for agitation. To characterize the enzymatic release of DAC, collagenase type IV is added to PBS at pH = 7.4. The DAC assay was performed by determining the UV absorbance of the solution to be tested at 255 nm and calculating the DAC UV absorbance from the DAC UV absorbance standard curve.

### 4.8 In Vitro Release of La^3+^ From GDLC Hydrogel Under Different pH Conditions

Initially, acetate‐ammonium acetate buffers of pH = 5.5, 6.5, and 7.4 were prepared. The concentration of La^3+^ in the buffer was measured at different time points using the azoarsenic III method. 0.5 mg/mL azoarsenic III solution and a pH = 2.8−3.0 chloroacetic acid buffer solution were prepared. Then, 10 µL of the solution to be measured was added to 40 µL of azoarsenic III solution and 200 µL of chloroacetic acid buffer. Subsequently, the UV absorbance at 630 nm was determined, and the content of La^3+^ was calculated based on the standard curve of UV absorbance.

### 4.9 Cell Viability Study

The cytotoxic effect of various compounds on 4T1 cells were assessed by using the MTT assay. Groups of Control, Gela NPs (10 mg/mL), DAC (8 mg/mL), LCNSs (La^3+^ 0.6 mm), La^3+^ (La^3+^ 0.6 mm), GLC (La^3+^ 0.6 mm), and GDLC hydrogel (La^3+^ 0.6 mm) were established. The materials were extracted using acetate buffer for 24 h and then diluted with RPMI‐1640 medium without serum and phosphate. The concentration of hydrogel extracts containing 0.6 mm La^3+^ was defined as 100%. 4T1 cells reached 90% confluence, after which the material was added and incubated for 24 h. At the end of the incubation period, 20 µL of MTT solution was added, and the mixture was incubated for 4 h in a cell incubator. Measurement of 490 nm absorbance after addition of DMSO. Cells were stained with calcein‐AM and propidium iodide (PI) after treatment with each group of materials, and the staining was then observed using an inverted fluorescence microscope.

### 4.10 Assessing the Specificity of Pyroptosis

Morphological observation of pyroptotic cells was conducted by localizing them using SYTOX Green. PBS, Gela NPs, DAC, LCNSs, La^3+^, GLC, and GDLC hydrogel (the GDLC hydrogel group was pretreated with 1 µm DAC for three days) were macerated in acetate buffer for 24 h and prepared in serum‐free and phosphate‐free RPMI‐1640 medium at the corresponding concentrations. The cells were incubated with SYTOX Green and various materials at 37°C for 30 min.

### 4.11 Mitochondrial Membrane Potential Staining

The mitochondrial membrane potential of 4T1 cells treated with different materials was determined using the JC‐1 kit.

### 4.12 Bioelectron Microscopy (Bio‐TEM) Observation

Cells treated with different materials were collected and fixed with a 5% glutaraldehyde aqueous solution for 24 h. The samples were fixed with osmium tetroxide solution, dehydrated, embedded, sectioned, and the cell sections were observed using a transmission electron microscope.

### 4.13 LDH and ATP Release

LDH and ATP released from pyroptosis were assayed using the LDH and the ATP Assay Kit (Beyotime, China).

### 4.14 Western Blot Analysis

Set control, Gela NPs, DAC, LCNSs, La^3+^, GLC, GDLC hydrogel groups. Cells treated with various materials were collected and lysed by adding cell lysate and a protease inhibitor for 30 min. The sample should be cooled to room temperature before being stored in a refrigerator at ‐20°C. Prepare a 12% electrophoretic separation gel and a 5% electrophoretic concentration gel. Electrophoresis was performed using a protein electrophoresis apparatus (WIX‐EP600, China) at 80 V for 30 min, 120 V for 75 min, and 250 mA electrotransfer for 2 h. The PVDF membrane was incubated with primary antibodies targeting the proteins of interest (anti‐GSDME antibody, anti‐cleaved‐Caspase 3 antibody, anti‐β‐actin antibody) at 4°C overnight. Add the appropriate amount of diluted secondary antibody and incubate it on a shaker at room temperature for 2 h. Imaging was performed using a protein imaging system (FluorChem E, USA).

### 4.15 In Vivo Biocompatibility Evaluation

To evaluate the in vivo drug release behavior and biocompatibility of GDLC hydrogels, we established a subcutaneous breast cancer model in mice: 1 × 10^6^ 4T1‐luc cells were injected subcutaneously into the back of BALB/c mice, and GDLC hydrogels were injected around the tumor once the tumor volume reached approximately 150 mm^3^. PBS, LCNSs, DAC, GLC, and GDLC hydrogels were injected subcutaneously into mice. The blood of mice was analyzed for routine blood tests and blood biochemistry on day 7 and day 14. The mice were euthanized and dissected to collect heart, liver, spleen, lung, and kidney tissues for H&E staining, as well as skin tissues for H&E staining and immunohistochemical staining (including Ki‐67, TNF‐α, IL‐6).

### 4.16 In Vivo Degradation of GDLC Hydrogel With La^3+^ Release

In order to characterize the degradation of GDLC hydrogel in the peritumoral environment, 100 µL of GDLC hydrogel was injected into the peritumoral area of 4T1 tumor model mice, and the same dose of GDLC hydrogel was injected at the same location in normal mice as a control. The mice were euthanized at different time points, and photographs were taken to record the degradation of GDLC hydrogel. The residual hydrogel was collected, and La^3+^ was detected using Inductively Coupled Plasma‐Mass Spectrometry (ICP‐MS).

### 4.17 Fluorescence Imaging of Mouse Tumors

100 µL of D‐luciferin potassium salt solution was injected intraperitoneally into 4T1‐Luc model mice, which were anesthetized and then allowed to wait for 5 min. Bioluminescence imaging of tumors was performed using an animal in vivo imager (Spectral Instruments, USA).

### 4.18 Flow Cytometry Analysis

After euthanasia, mice are dissected, and tissues such as tumors, spleen, and lymph nodes are collected for analysis. Surface mucus is rinsed off with FACS buffer, and tissues are digested with a medium containing collagenase IV. The antibody was diluted with FACS buffer, and the staining was incubated for 30 min on ice. For intracellular staining: After completing extracellular staining, cells were fixed, and their membranes were ruptured using the TranscriptionFactor Staining Buffer Set (Thermo, USA). Diluted flow antibody in FACS buffer was added, and staining was incubated for 30 min on ice. Cells were analyzed using a flow cytometer (BD FACSymphony, USA).

### 4.19 Measurement of Serum Inflammatory Factor

Blood from different groups (Control, La^3+^, LCNSs, DAC, GLC, GDLC hydrogel, aTIGIT, and GDLC hydrogel + aTIGIT) of mice was collected. Serum inflammatory factors in mice were detected using TNF‐α, IL‐6, and IFN‐γ ELISA kits (Wuhan Colorful Gene Biotech Co., Ltd, China).

### 4.20 Evaluation of Anti‐Tumor Effects

All animal experimentation protocols received approval from the Institutional Animal Care and Use Committee at Hefei University of Technology (No. HFUT20230326001).

The mouse breast cancer model was established by subcutaneously injecting 1 × 10^6^ luciferase‐tagged 4T1 cells (4T1‐Luc) into 5−6‐week‐old female BALB/c mice. This was done to confirm the anti‐tumor effect of GDLC hydrogel and its components in a murine subcutaneous tumor model, as well as to evaluate the therapeutic impact of combined immune checkpoint inhibitors. The following experimental groups were set up: Control (50 µL of saline), LaCl_3_‐7H_2_O (300 µg), LCNSs (2.5 mg), DAC (200 µg), GLC (50 µL), GDLC hydrogel (50 µL), aTIGIT (intraperitoneal injection of 200 µg each time for a total of 4 injections once every 3 days), and GDLC hydrogel (50 µL) + aTIGIT. Upon reaching a tumor volume of 100 mm^3^ in the murine subcutaneous breast cancer model, samples from each experimental group were administered into the peri‐tumoral region through subcutaneous injection. Tumor volume and mouse survival were recorded. The mice were euthanized and dissected on day 14. Serum was collected to determine inflammatory factors, while tumor tissues were obtained for immunohistochemical analysis (including Ki‐67, HMGB1, caspase‐3, and GSDME), immunofluorescence analysis (CD8 and CD49b), and flow cytometric analysis.

### 4.21 Distant Tumor Suppressor Effect

The double tumor model was established using 5−6‐week‐old female BALB/c mice. The following experimental groups were set up: Control group (50 µL of saline), LCNSs (2.5 mg), DAC (200 µg), GDLC hydrogel (50 µL), aTIGIT (intraperitoneal injection of 200 µg each time, a total of 4 injections once every 3 days), and GDLC hydrogel (50 µL) + aTIGIT. Upon reaching a tumor volume of 100 mm^3^ in the mice, the sample groups were administered injections around the periphery of the right tumor. Tumor volume and mouse survival were recorded. Mice were euthanized and dissected on day 14. Tumors from both ends of each group were collected for flow cytometric analysis.

### 4.22 Preventing Postoperative Tumor Recurrence

The postoperative model was established using 5−6‐week‐old female BALB/c mice. The following experimental groups were set up: Control group (50 µL of saline), LCNSs (2.5 mg), DAC (200 µg), GDLC hydrogel (50 µL), aTIGIT (intraperitoneal injection of 200 µg each time, a total of 4 injections once every 3 days), and GDLC hydrogel (50 µL) + aTIGIT. After the surgical resection of the tumors, materials from different subgroups were applied to the postoperative wounds with 50 µL on the trauma. Mice were euthanized and dissected on day 21. Lungs of mice were collected for hematoxylin and eosin (H&E) staining, and tumors were collected for immunofluorescence analysis (CD8 and CD49b).

### 4.23 Long‐Term Tumor Immune Memory Effects

Postoperative model mice were treated with GDLC hydrogel (50 µL) + aTIGIT (intraperitoneal injection of 200 µg/dose, 4 injections administered once every 3 days). The surviving mice were reimplanted with 1 × 10^6^ 4T1‐Luc cells, and the tumor volume was recorded along with the survival rate of the mice. On day 60, a subset of mice was euthanized, and their spleen and lymph nodes were harvested for flow cytometric analysis to assess the populations of central memory T cells and effector memory T cells.

### 4.24 RNA Sequencing

RNA was extracted from 4T1 cells treated with the control group and GDLC hydrogel (with DAC 1 µm, 3 days) using Trizol and stored at −80°C. Shanghai Personal Biotechnology Co., Ltd. was commissioned to perform RNA sequencing.

### 4.25 Statistics Analysis

All experimental data in this study were recorded as mean ± standard deviation (SD). One‐way ANOVA with Tukey's post‐hoc test was applied to evaluate the statistically significant difference of multiple group data. Two‐sided paired t‐test was applied to evaluate the statistically significant difference of two group data. The p‐value was denoted by ^*^ for *p* < 0.05, ^**^ for *p* < 0.01, and ^***^ for *p* < 0.001.

## Author Contributions

**Haitao Wu**: Writing – review and editing, methodology. **Yaoyu Hu**: investigation. **Xuan Zhang**: investigation. **Zhengbao Zha**: conceptualization, writing – review and editing, supervision, funding acquisition. **Yang Hong**: methodology, writing – original draft, investigation. **Qian Chen**: investigation. **Weitao Wang**: investigation.

## Conflicts of Interest

The authors declare no conflicts of interest.

## Supporting information




**Supporting file**: advs76966‐sup‐0001‐SuppMat.docx.

## Data Availability

The data that support the findings of this study are available from the corresponding author upon reasonable request.
