# Supplementary material for: Lanthanum Carbonate Nanosheets Based Colloidal Hydrogel for Modulating Innate & Adaptive Tumor Immunotherapy by Augmented Cellular Pyroptosis
Source: Adv Sci (Weinh). 2026 Aug 3:e76966. Online ahead of print. doi: 10.1002/advs.76966 (PMC13430939; doi:10.1002/advs.76966)
Supplement: Supplementary file 1 — Supporting file: advs76966‐sup‐0001‐SuppMat.docx. [file ADVS-9999-e76966-s001.docx]

Lanthanum carbonate nanosheets based colloidal hydrogel for modulating innate & adaptive tumor immunotherapy by augmented cellular pyroptosis

Yang Hong^#^, Haitao Wu^#^, Weitao Wang, Xuan Zhang, Qian Chen, Yaoyu Hu, Zhengbao Zha*

[*] Prof. Z. B. Zha, Y. Hong

School of Medicine and Health, Zhengzhou Advanced Research Institute of Harbin Institute of Technology, Zhengzhou 450003, China

E-mail: [zbzha@hit.edu.cn](mailto:zbzha@hit.edu.cn)

[*] Prof. Z. B. Zha, Y. Hong, W. T. Wang, X. Zhang, Q. Chen, Y. Y. Hu

School of Food and Biological Engineering, Hefei University of Technology, Hefei, 230009, China

E-mail: zbzha@hfut.edu.cn

[*] Dr. H. T. Wu

School of Biomedical Engineering, Anhui Medical University, Hefei, 230032, China

^#^ These authors contributed equally to this work.


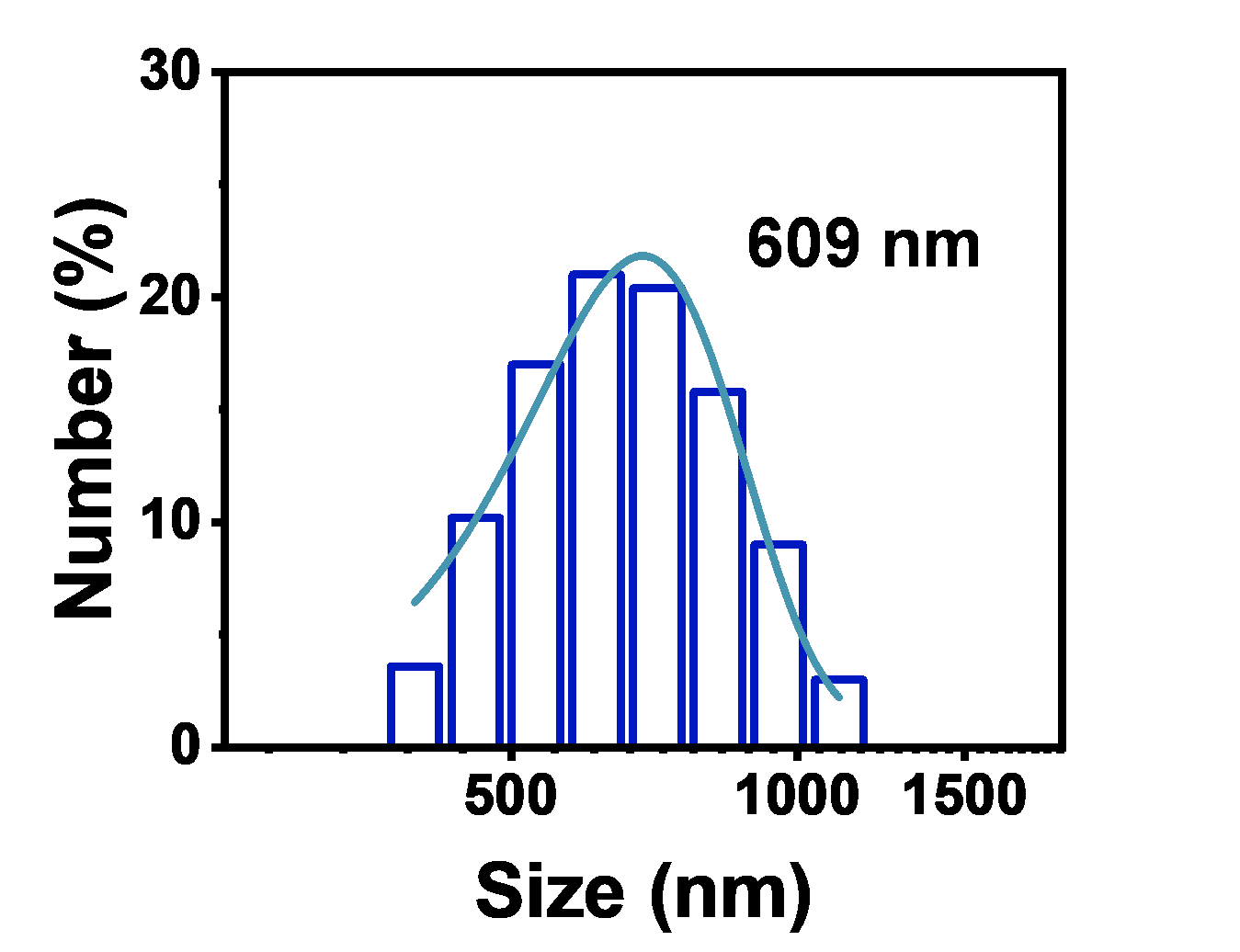


**Figure S1**. Hydrated particle size distribution of LCNSs. (Average hydrated particle size: 609 nm).


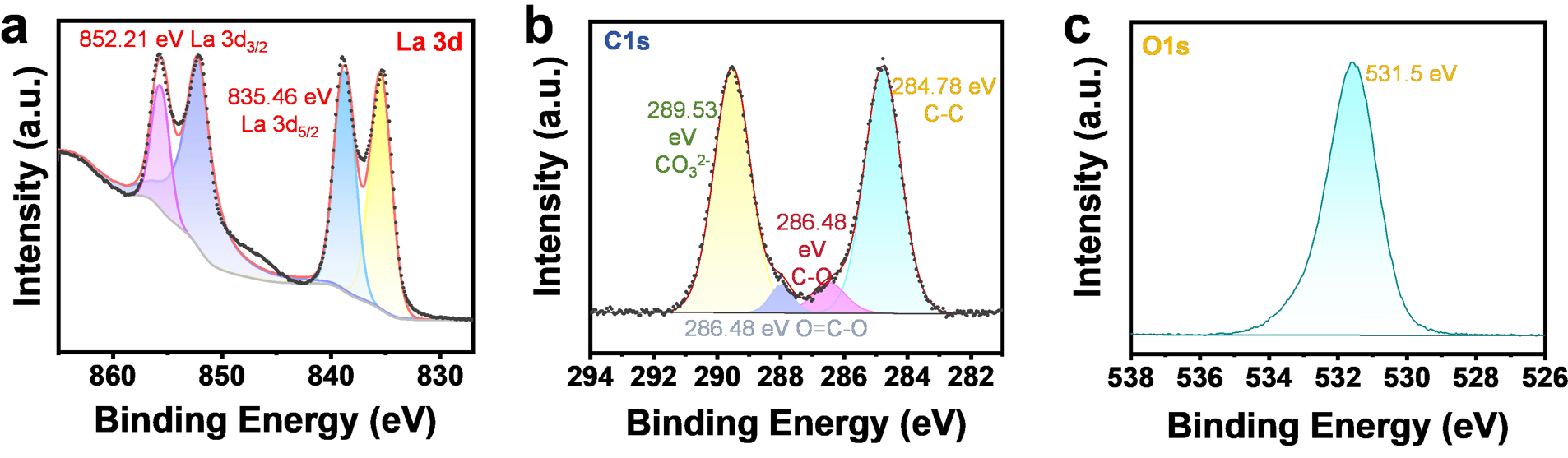


**Figure S2**. XPS spectra of LCNSs. (a) La 3d spectra. (b) C 1s spectra. (c) O 1s spectra.


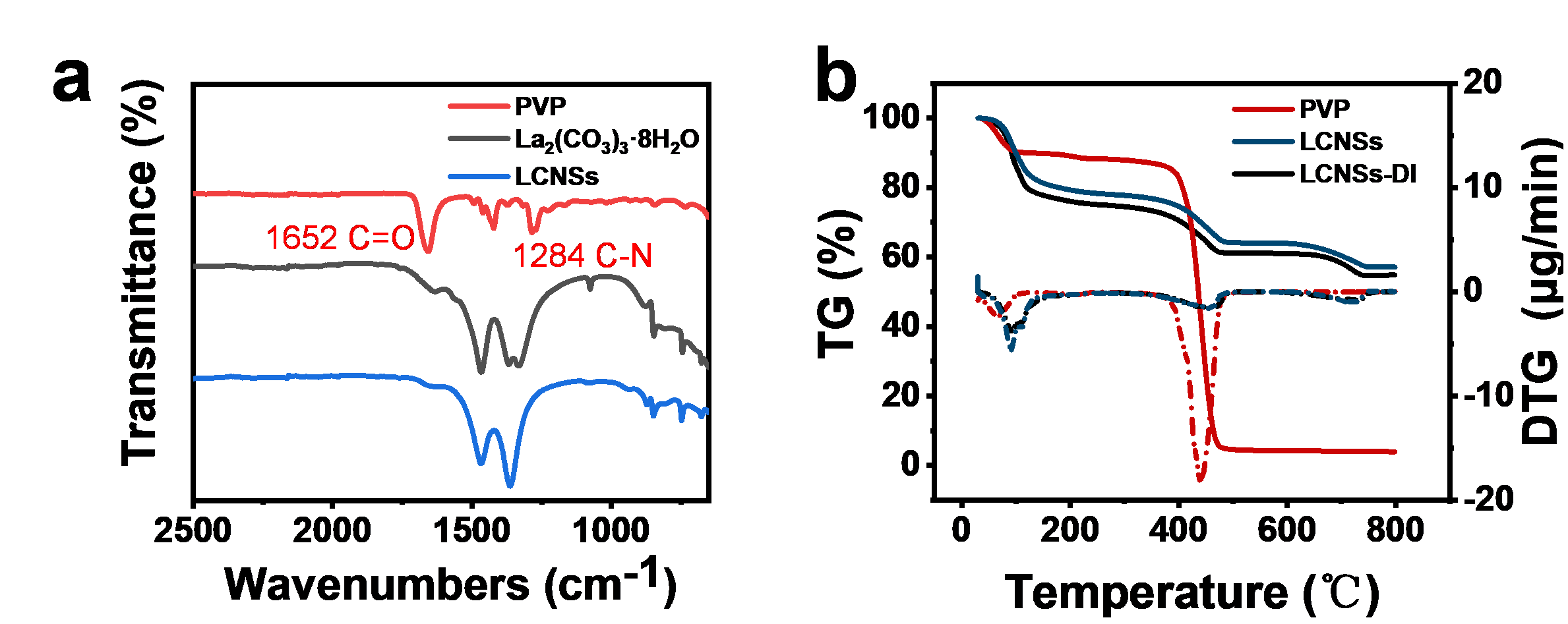


**Figure S3.** No PVP in LCNSs. (a) Fourier Transform infrared (FTIR) spectra analysis of PVP, LCNSs, and La_2_(CO_3_)_3_ 8H_2_O. (b) TG and DTG analysis of PVP, LCNSs prepared in PVP, and LCNSs prepared in DI water (LCNSs-DI).


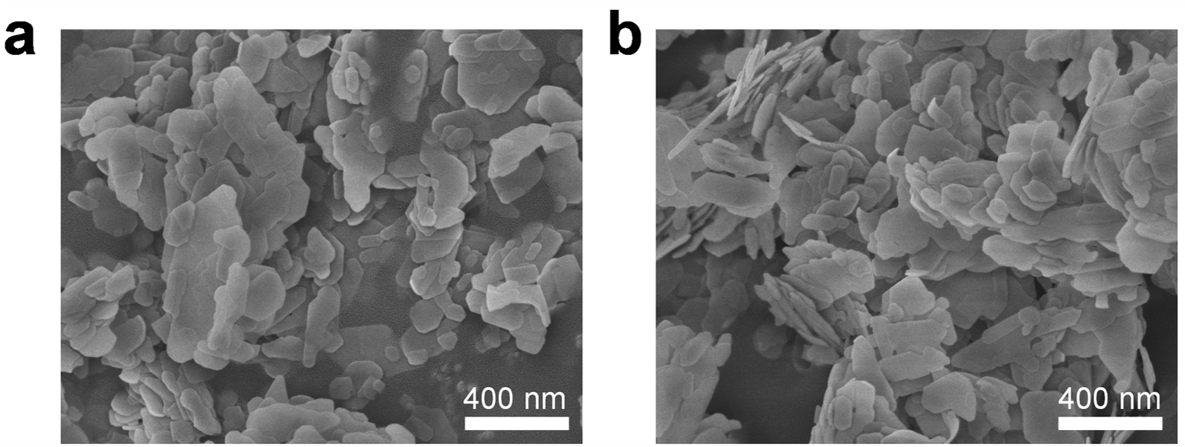


**Figure S4**. Characterization of LCNSs and BSA/LCNSs. SEM image of (a) LCNSs and (b) BSA/LCNSs. (Scale bar: 400 nm).


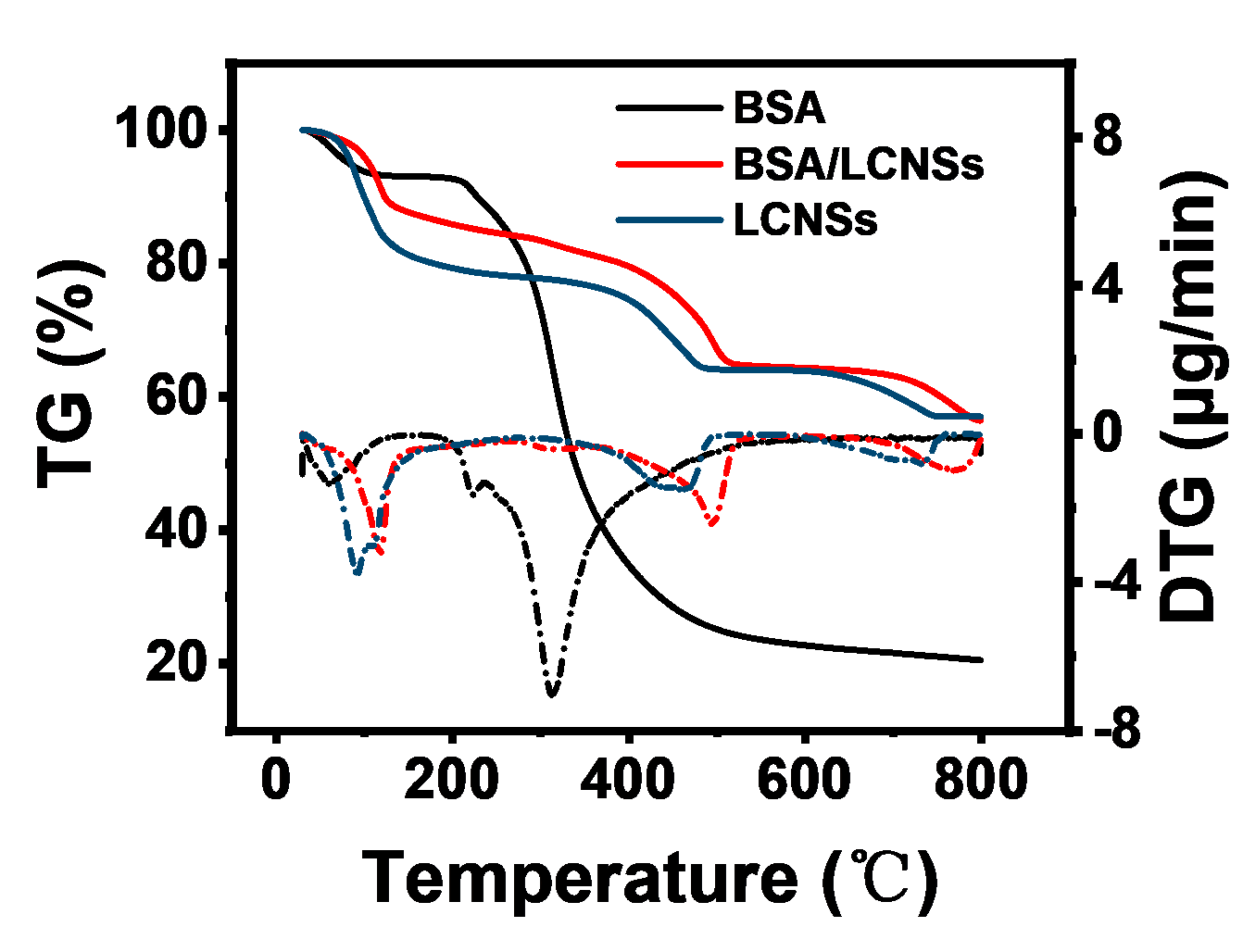


**Figure S5**. Thermogravimetric (TG) and Differential Thermogravimetric (DTG) analysis of BSA, LCNSs, and BSA/LCNSs.


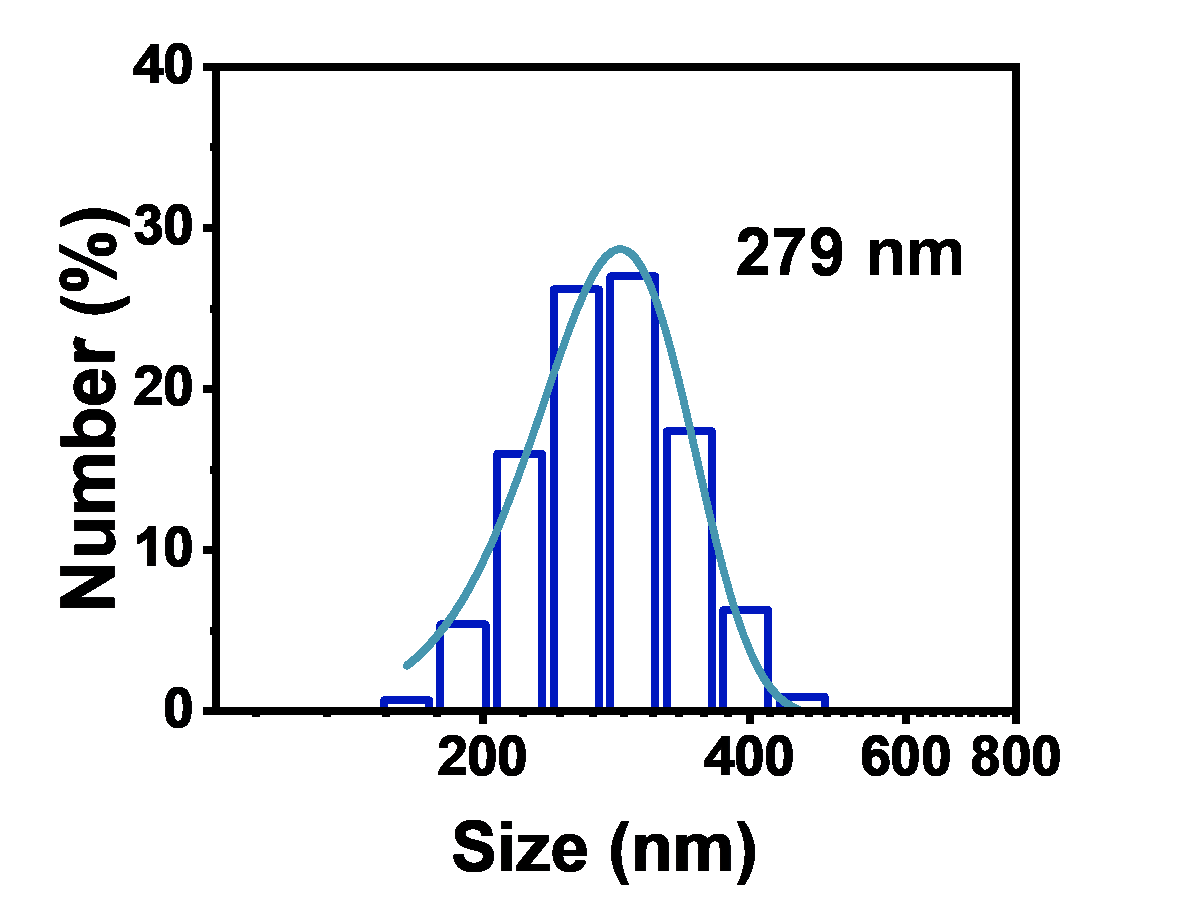


**Figure S6**. Hydrated particle size distribution of Gela NPs. (Average hydrated particle size: 279 nm).


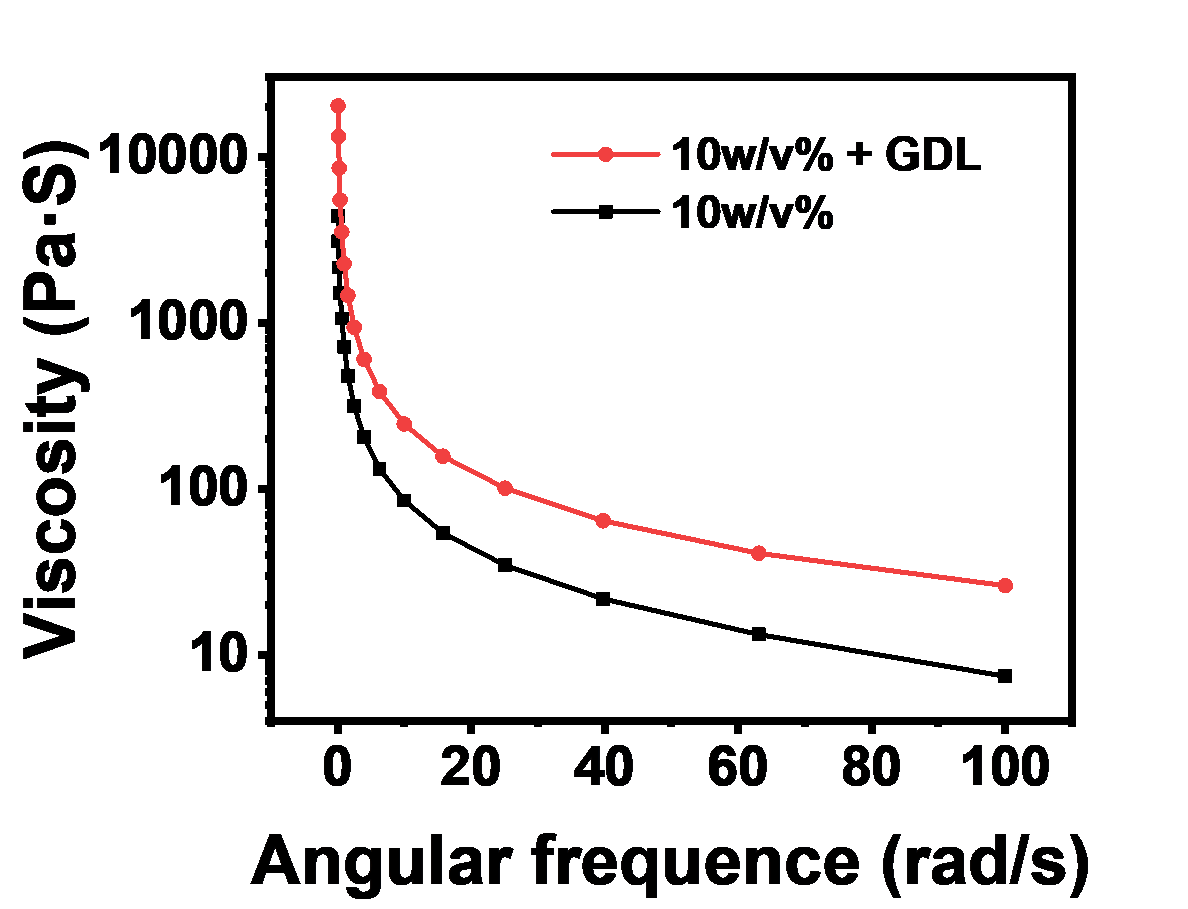


**Figure S7**. Effect of GDL on the shear thinning properties of 10 w/v% nanocolloidal hydrogels.


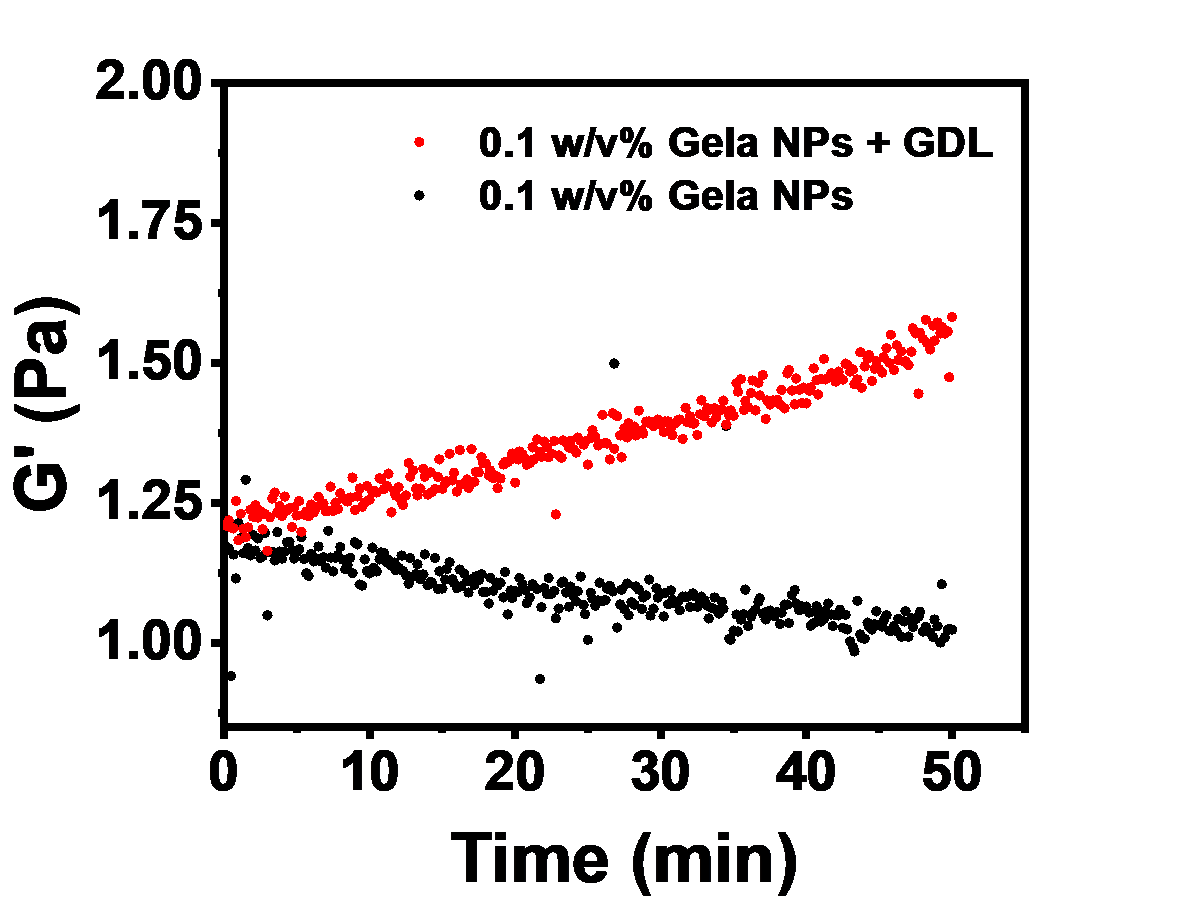


**Figure S8**. Effect of GDL on the gel-forming properties of Gela NPs.


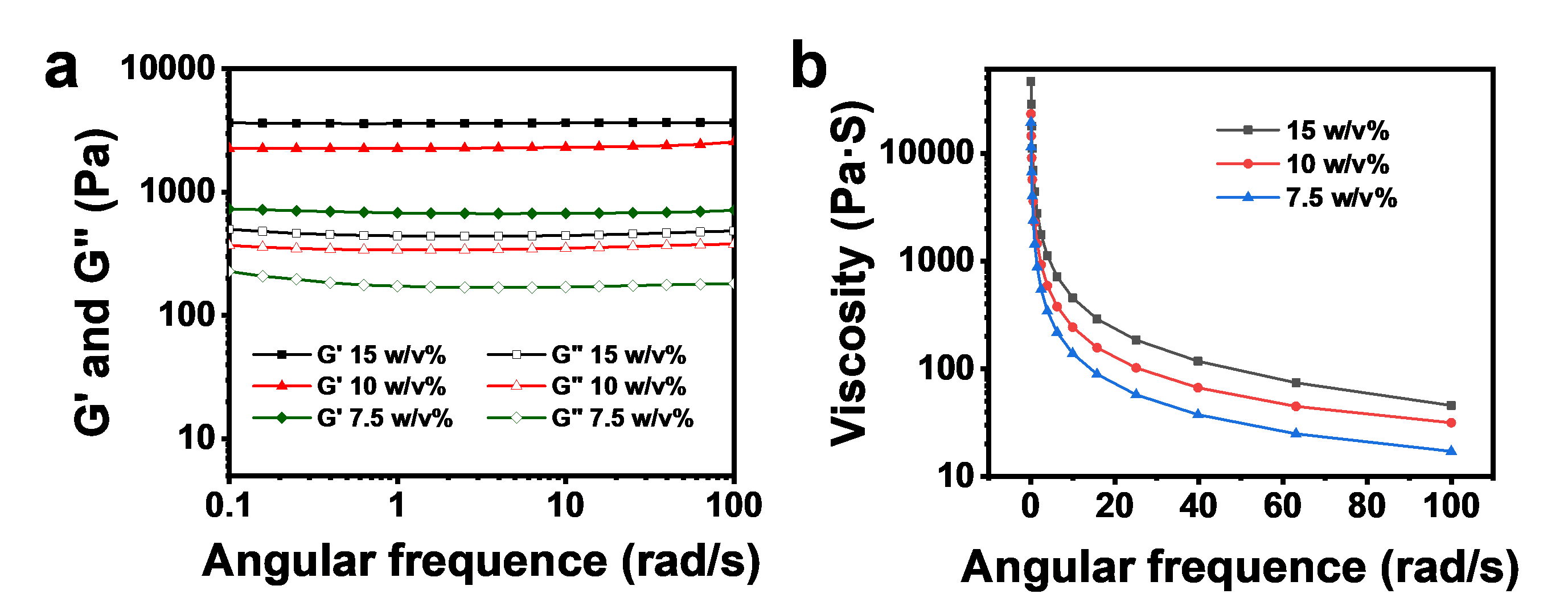


**Figure S9.** Influence of solid content on colloidal hydrogels. (a) Rheological analysis of GDLC with mass concentrations of 7.5, 10, and 15 w/v% at a 1:1 ratio of LCNSs to Gela NPs. (Frequency: 0.1~100 Hz). (b) Rheological analysis of the shear thinning properties of GDLC with mass concentrations of 7.5, 10, and 15 w/v% at a 1:1 ratio of LCNSs to Gela NPs.


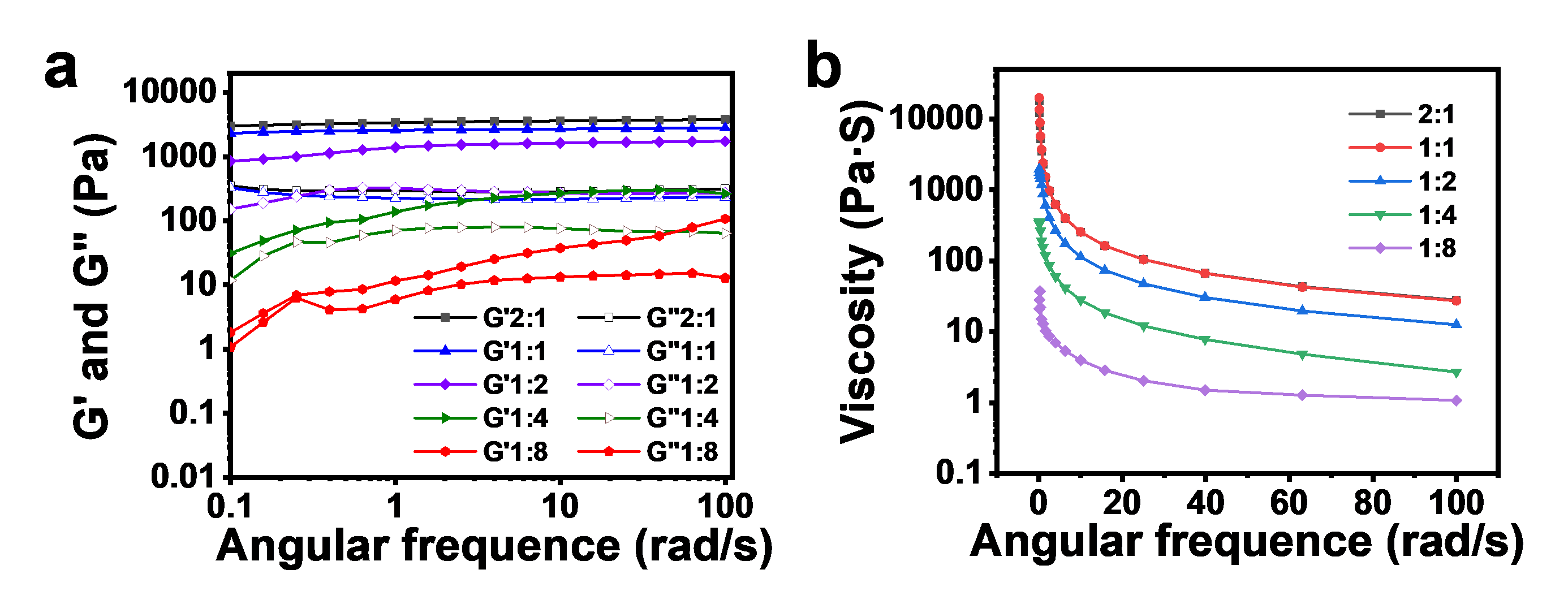


**Figure S10.** Influence of the ratio of LCNSs to Gela NPs on colloidal hydrogels. (a) Rheological analysis of GDLC with different ratios of LCNSs to Gela NPs at a mass concentration of 10 w/v%. (Frequency: 0.1~100 Hz). (b) Rheological analysis of the shear thinning properties of GDLC with different ratios of LCNSs to Gela NPs at a mass concentration of 10 w/v%.


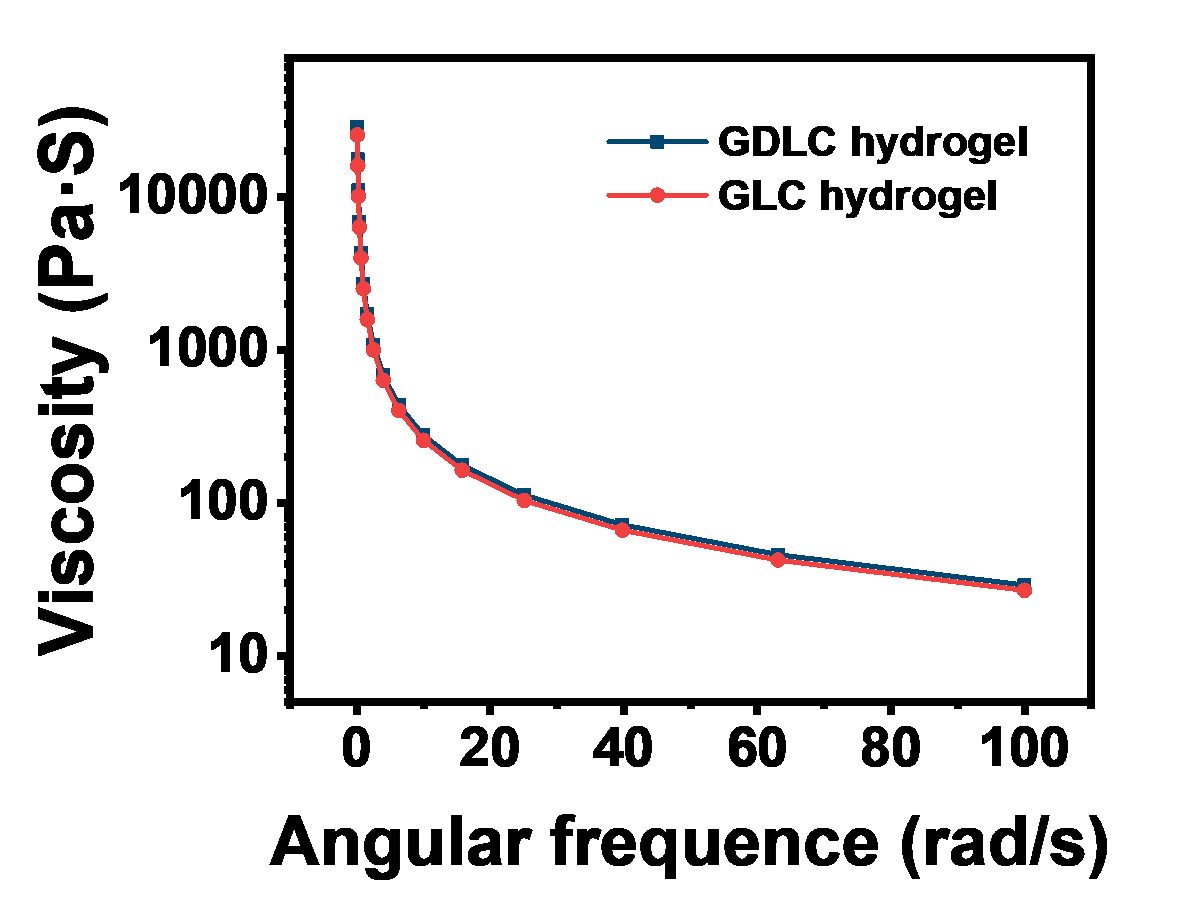


**Figure S11**. Influence of DAC on the shear thinning properties of 10 w/v% nanocolloidal hydrogels.


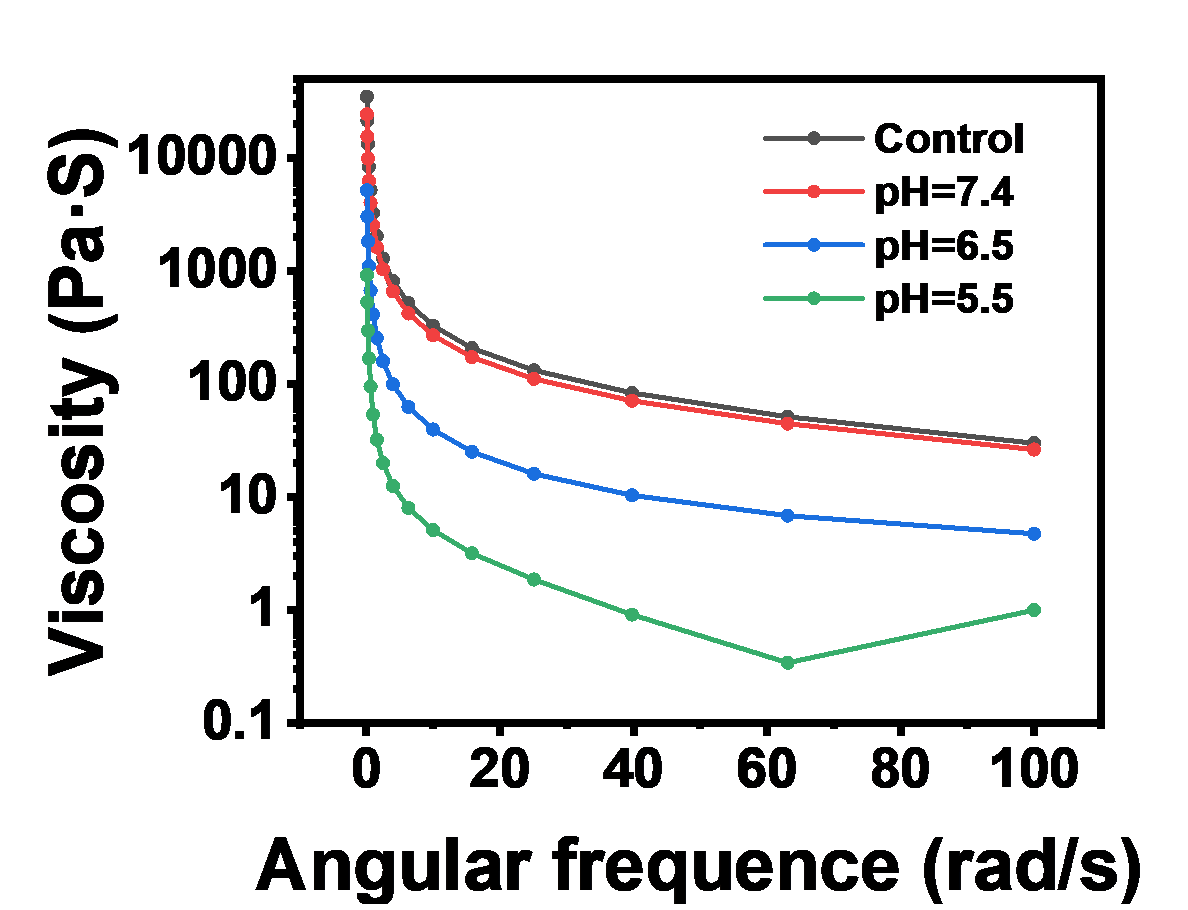


**Figure S12**. Rheological analysis of shear thinning properties of GDLC under different pH conditions.


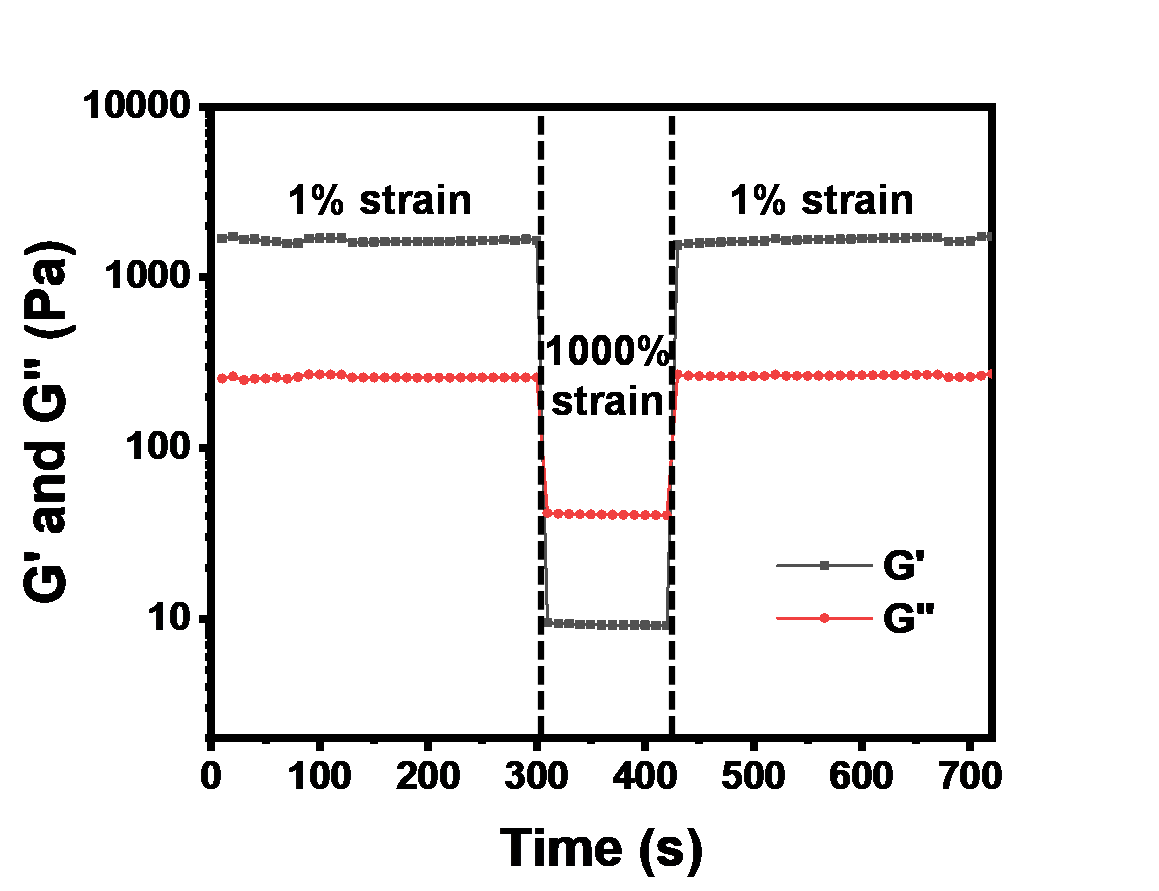


**Figure S13**. Rheological analysis of self-healing performance of GDLC.


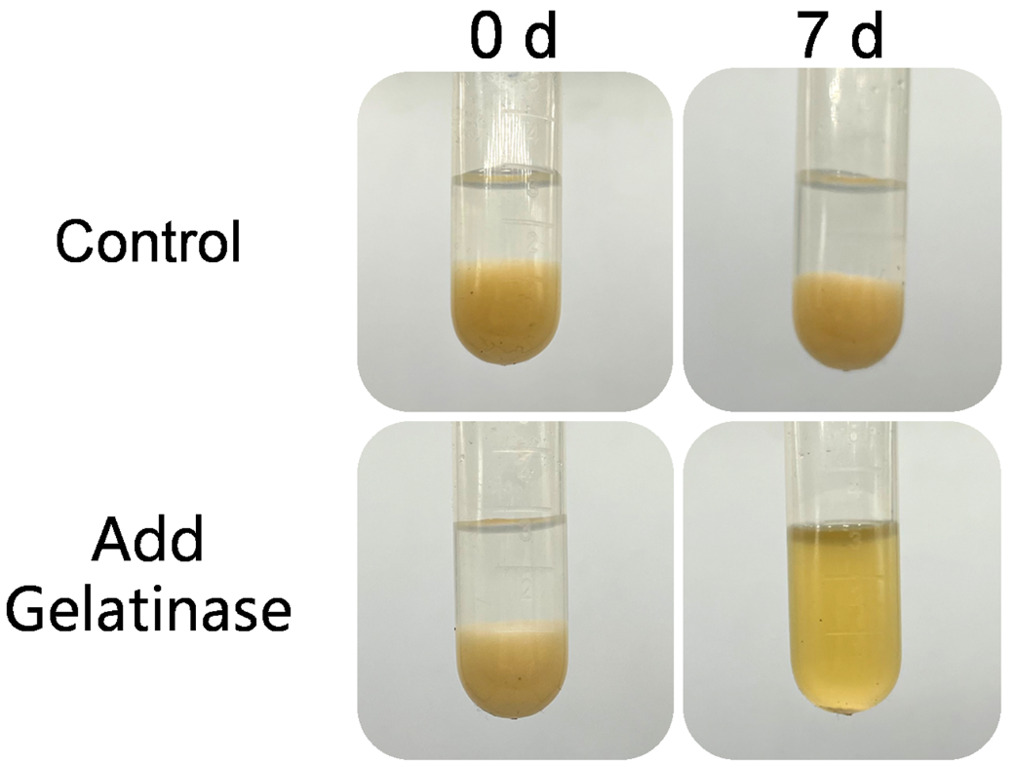


**Figure S14**. Enzymatic degradation of Gela NPs.


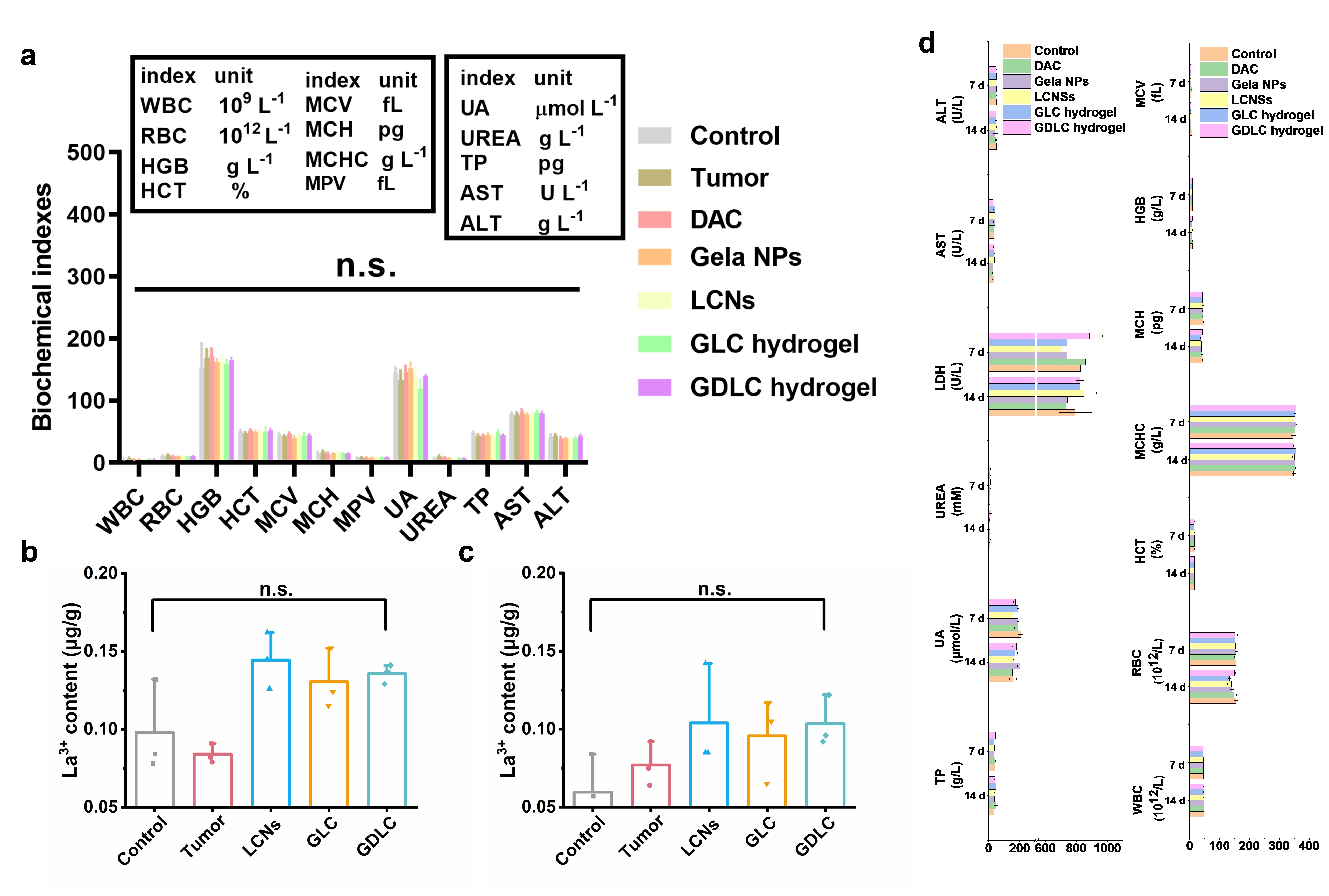


**Figure S15**. (a) Hematological analysis of tumor bearing mice in each experimental group; (b) Residual La^3+^ content in the tumor bearing mice liver and (c) kidney. (d) Blood biochemistry and routine blood parameters of normal mice treated with each experimental group.


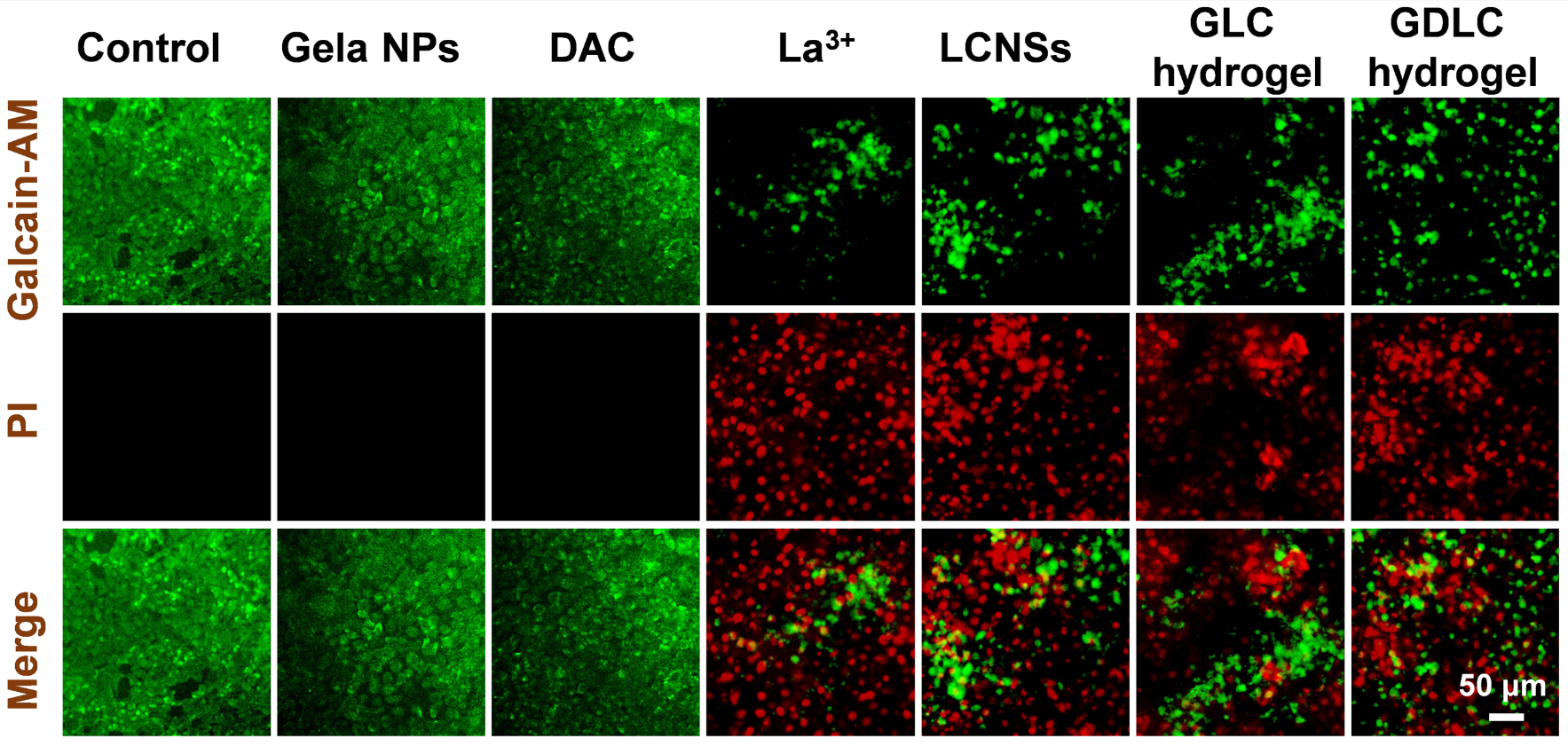


**Figure S16**. Galcain-AM/PI staining of 4T1 cells after different treatments under mildly acidic conditions (pH=7.4).
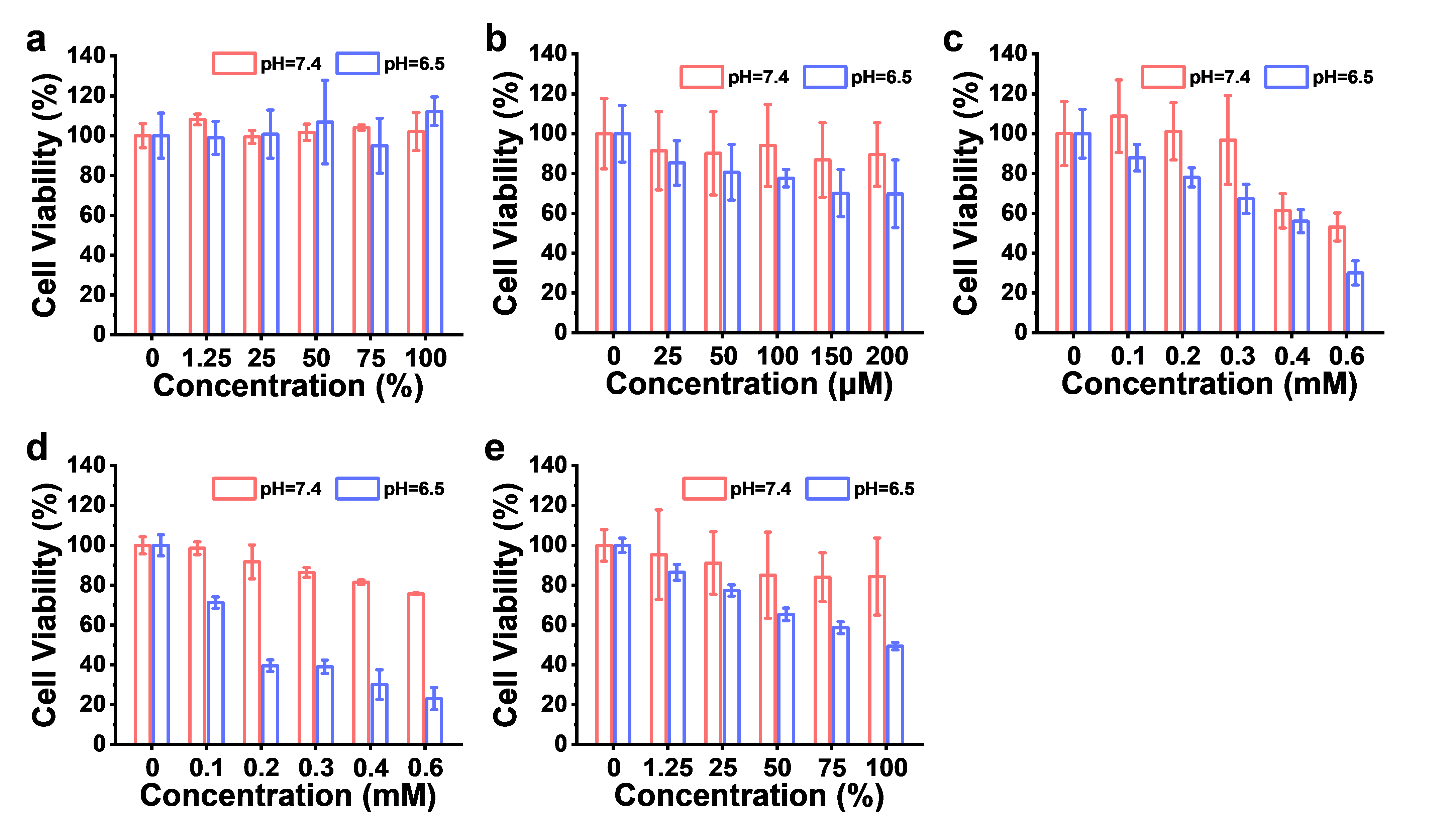


**Figure S17.** Cytotoxicity of 4T1 cells under various treatments. Cytotoxicity of 4T1 cells after incubation with different concentrations of (a) Gela NPs extracts, (b) DAC, (c) La^3+^, (d) LCNSs, and (e) GLC hydrogel extracts at pH=6.5 and 7.4.


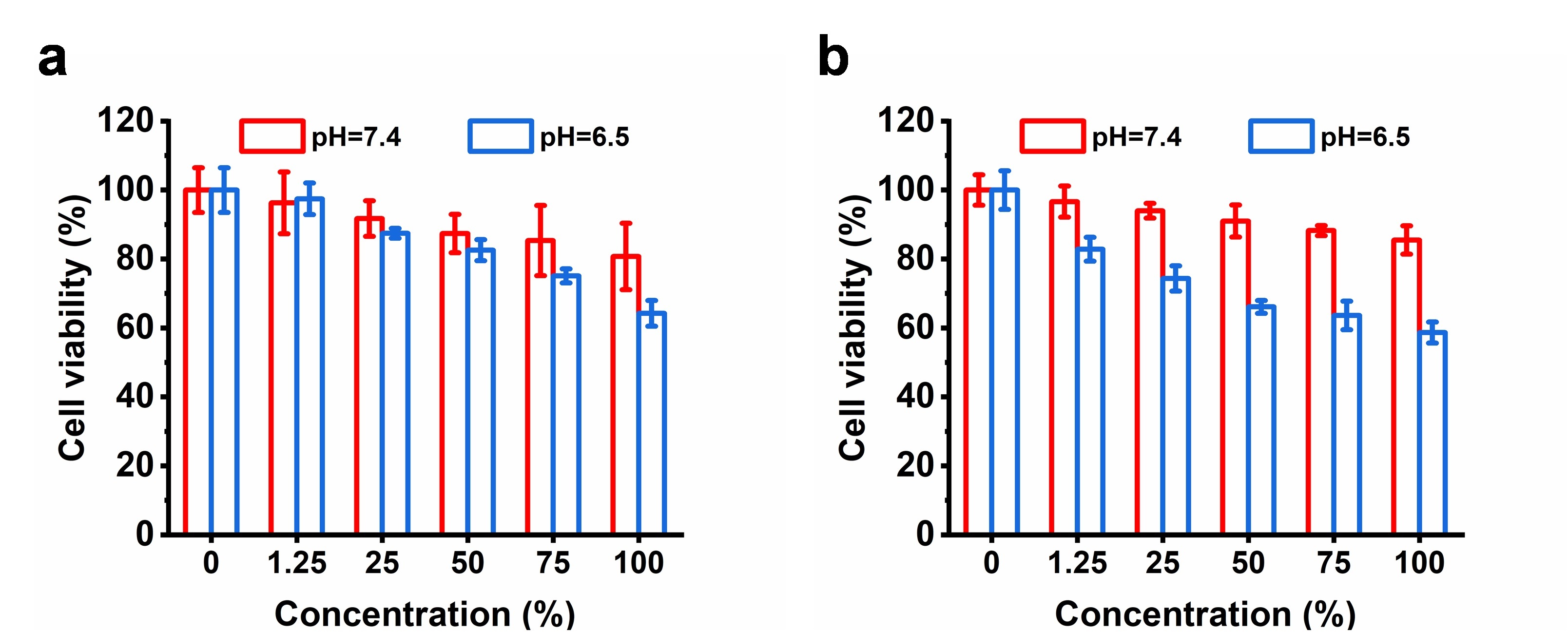


**Figure S18.** Cell viabilities of (a) AML12 and (b) RAW264.7 cells after incubation with different concentrations of GDLC hydrogel extracts at pH 6.5 and 7.4.


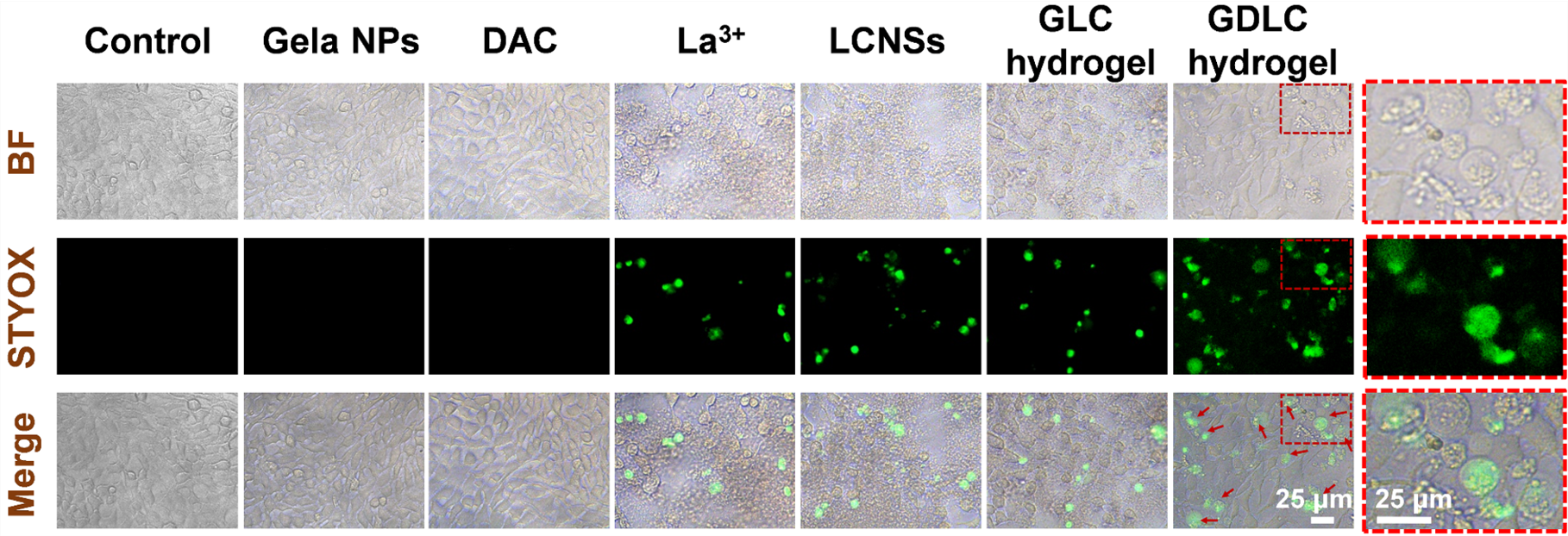


**Figure S19**. STYOX staining of 4T1 cells after various treatments at pH=7.4.


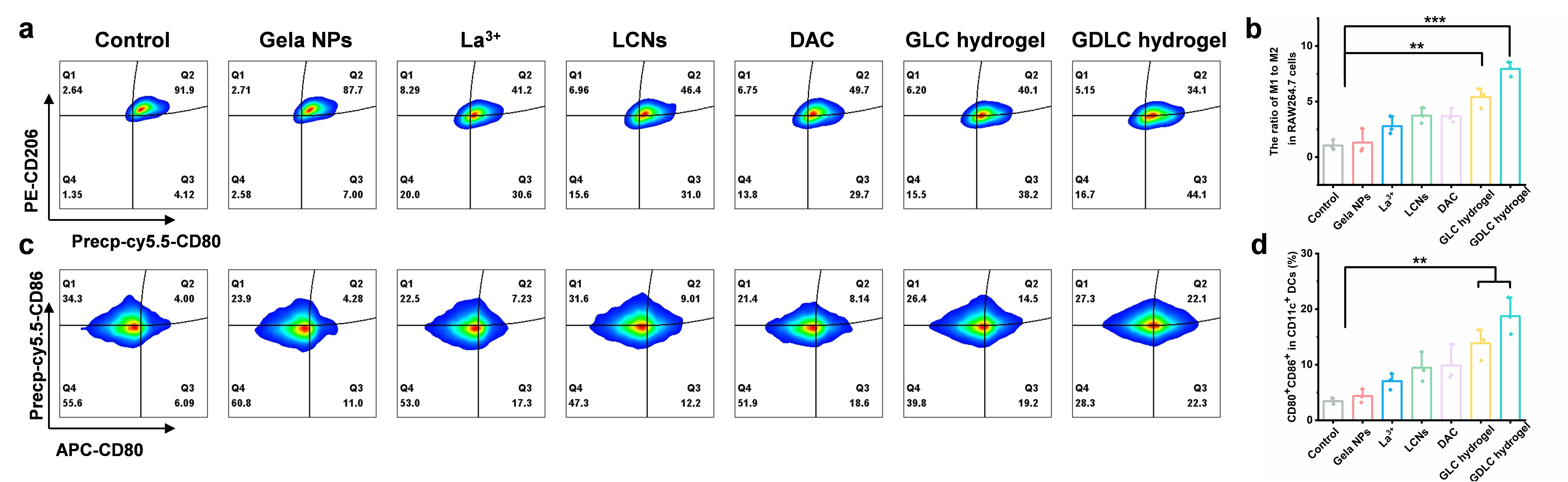


**Figure S20**. Flow cytometry analysis of (a) polarization of RAW264.7 cells and (c) BMDCs maturation. Quantitative results of (b) ratio of M1/M2 and (d) matured BMDCs (n = 3). The experimental groups included Control, Gela NPs (10 mg/mL), DAC (8 mg/mL), LCNSs (La^3+^ 0.6 mM), La^3+^ (0.6 mM), GLC (La^3+^ 0.6 mM), and GDLC hydrogel (La^3+^ 0.6 mM).


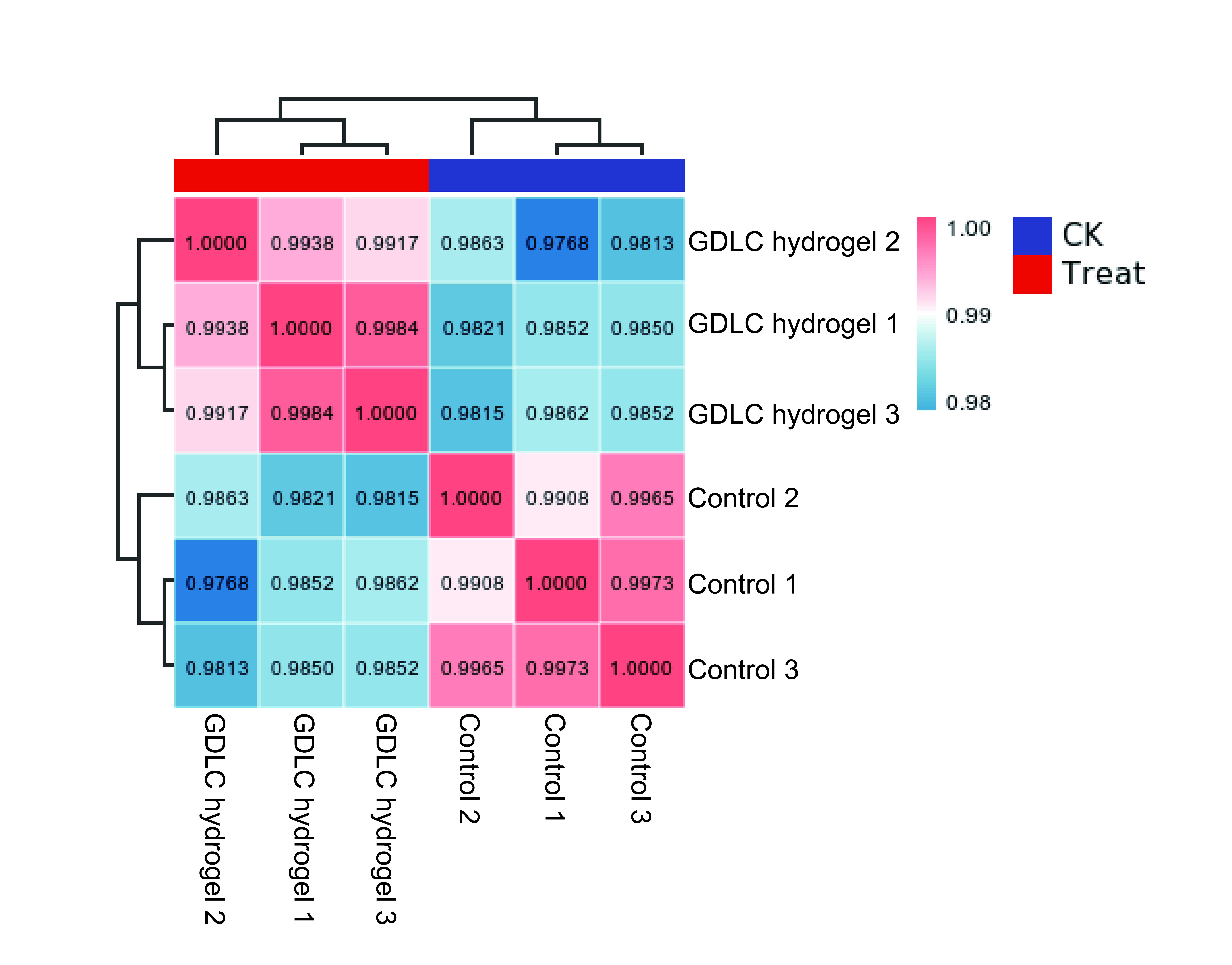


**Figure S21**. Unsupervised hierarchical clustering of RNA-Seq data from control and GDLC groups.


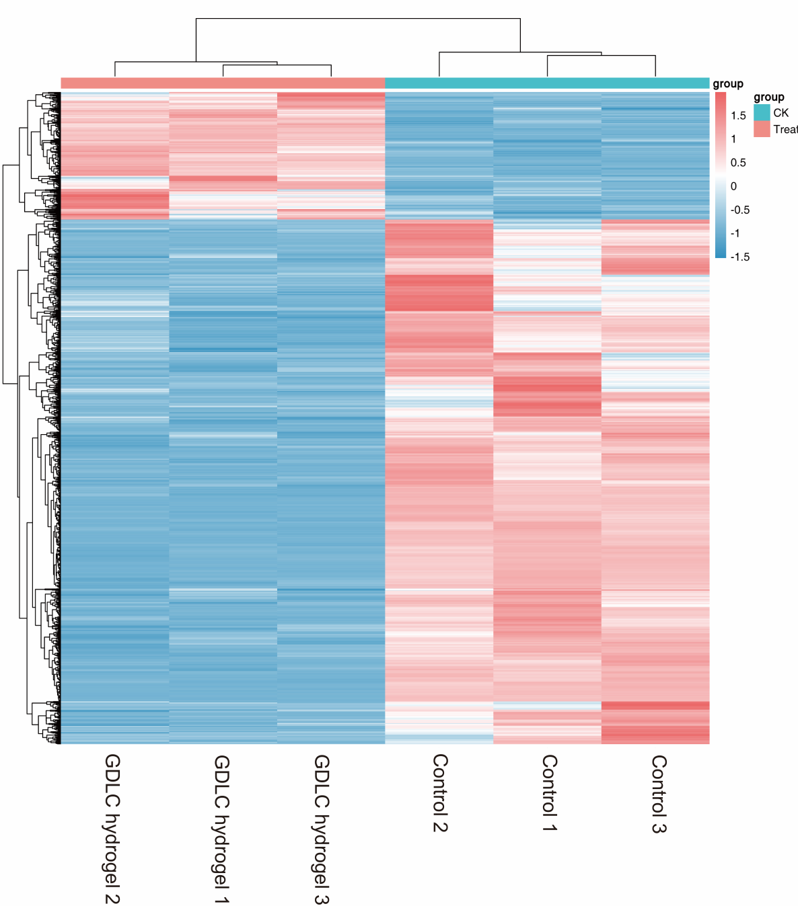


**Figure S22**. Cluster plot of differentially expressed genes (DEGs) between GDLC and control group.


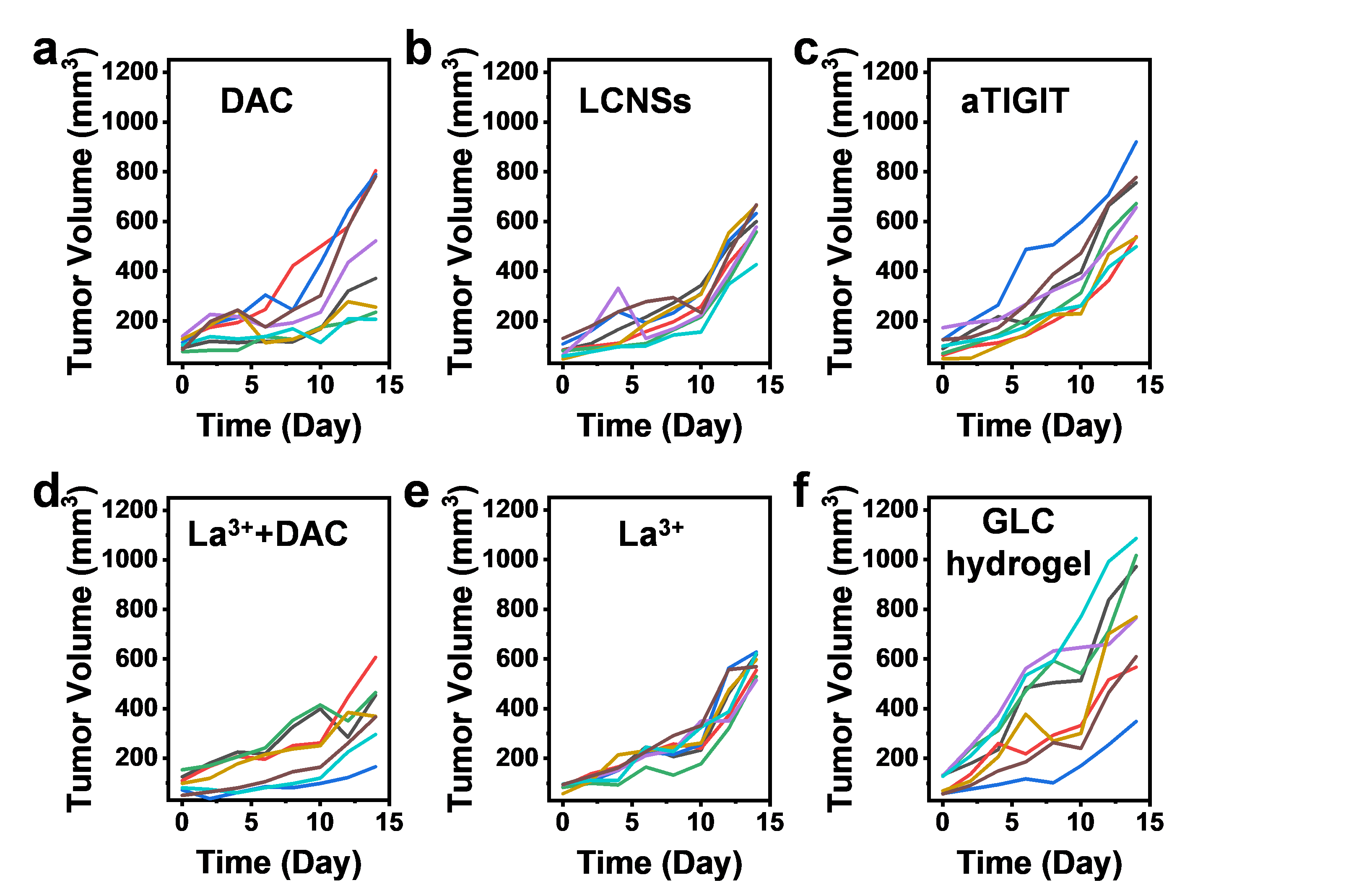


**Figure S23.** Tumor growth curves of 4T1-tumor-bearing mice with different treatments. Individual tumor growth curves of 4T1-tumor-bearing mice in (a) DAC, (b) LCNSs, (c) aTIGIT, (d) La^3+^+DAC, (e) La^3+^, and (f) GLC groups (*n*=8).


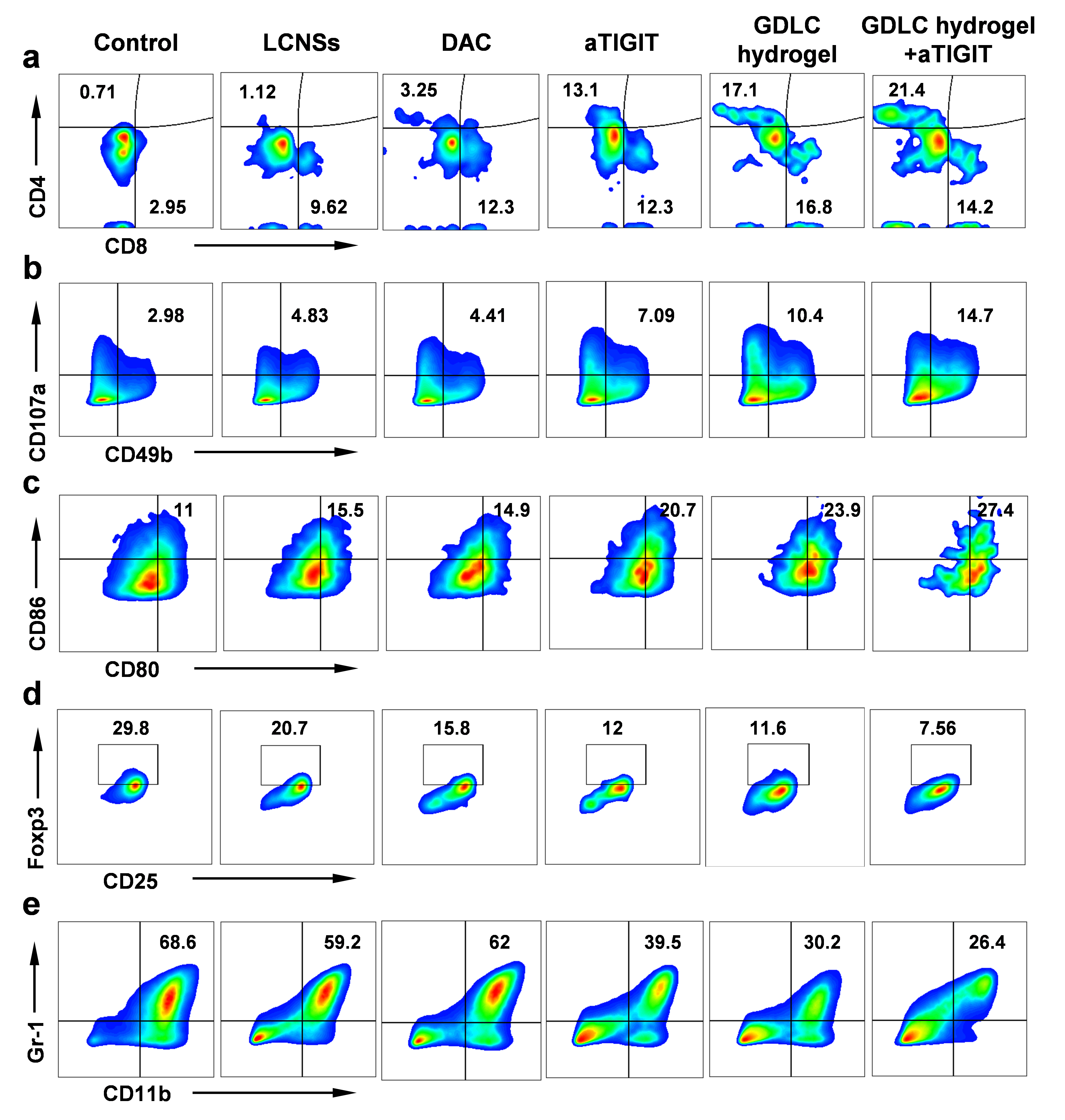


**Figure S24.** Flow cytometric analysis of anti-tumor immune activation. Flow cytometric analysis of (a) CD4^+^ and CD8^+^ T cells, (b) NK cells, (c) DCs, (d) Tregs, and (e) MDSC in the tumors of 4T1-tumor-bearing mice in different treatment groups.


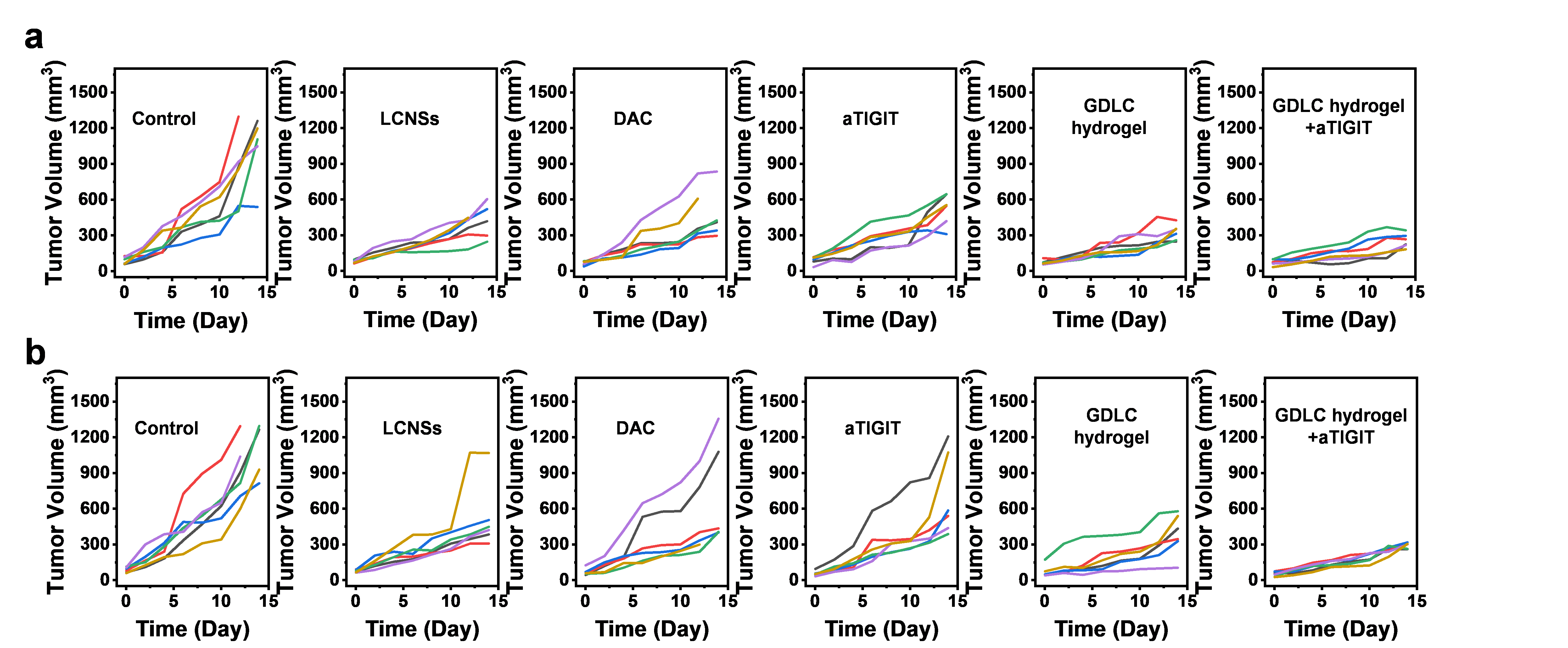


**Figure S25.** Inhibition of distal tumors. (a) Individual primary tumor growth curves, and (b) distant tumor growth curves of 4T1-tumor-bearing mice in different groups (*n*=8).


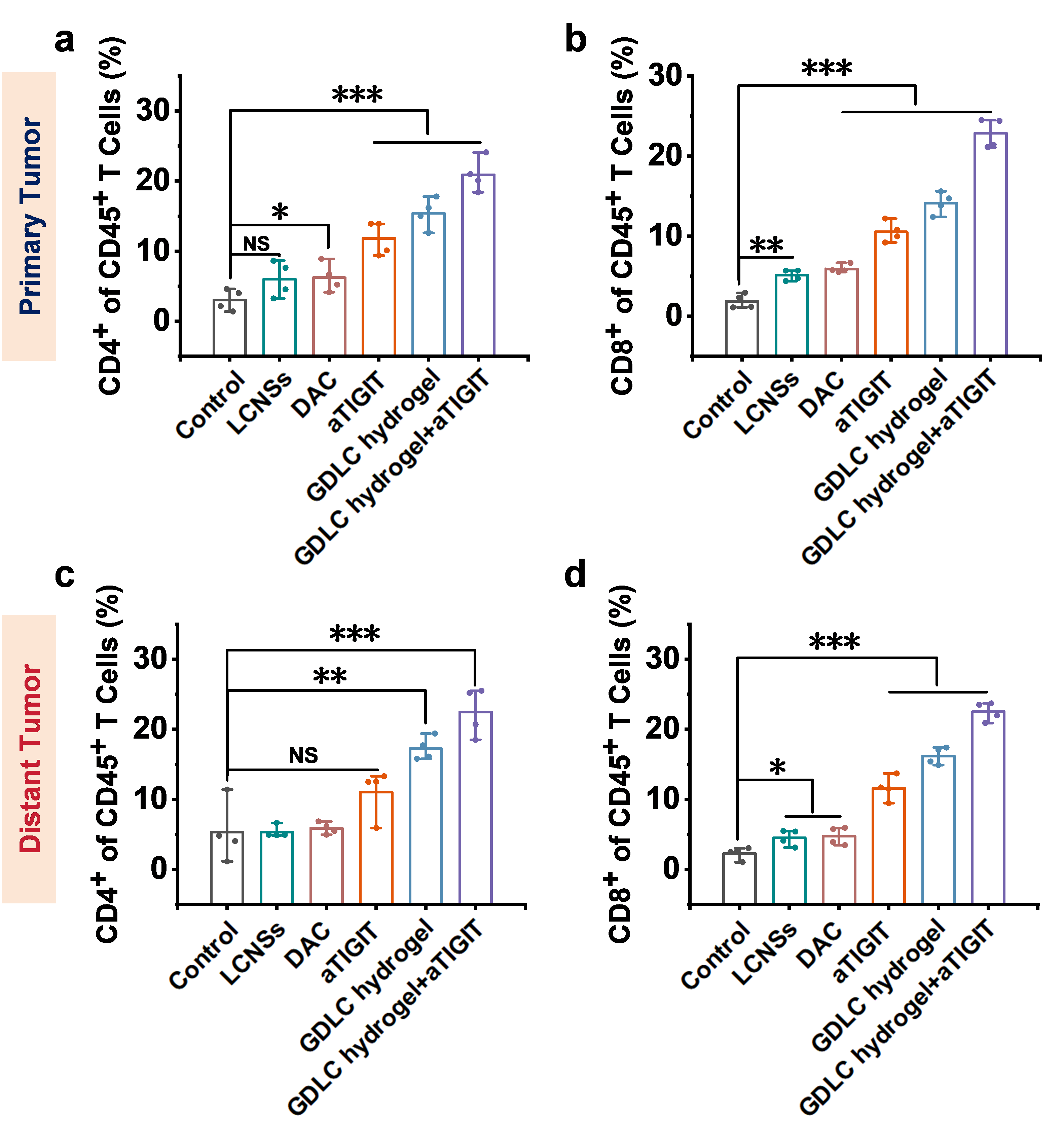


**Figure S26.** Flow cytometric analysis of CD4^+^ and CD8^+^ T cells. Flow cytometric analysis of (a) CD4^+^ and (b) CD8^+^ T cells in the primary tumors of 4T1-tumor-bearing mice in different treatment groups (*n*=4). Flow cytometric analysis of (c) CD4^+^ and (d) CD8^+^ T cells in the distant tumors of 4T1-tumor-bearing mice in different treatment groups (*n*=4). NS: no significant difference, **p* < 0.05, ***p* < 0.01, and ****p* < 0.001.


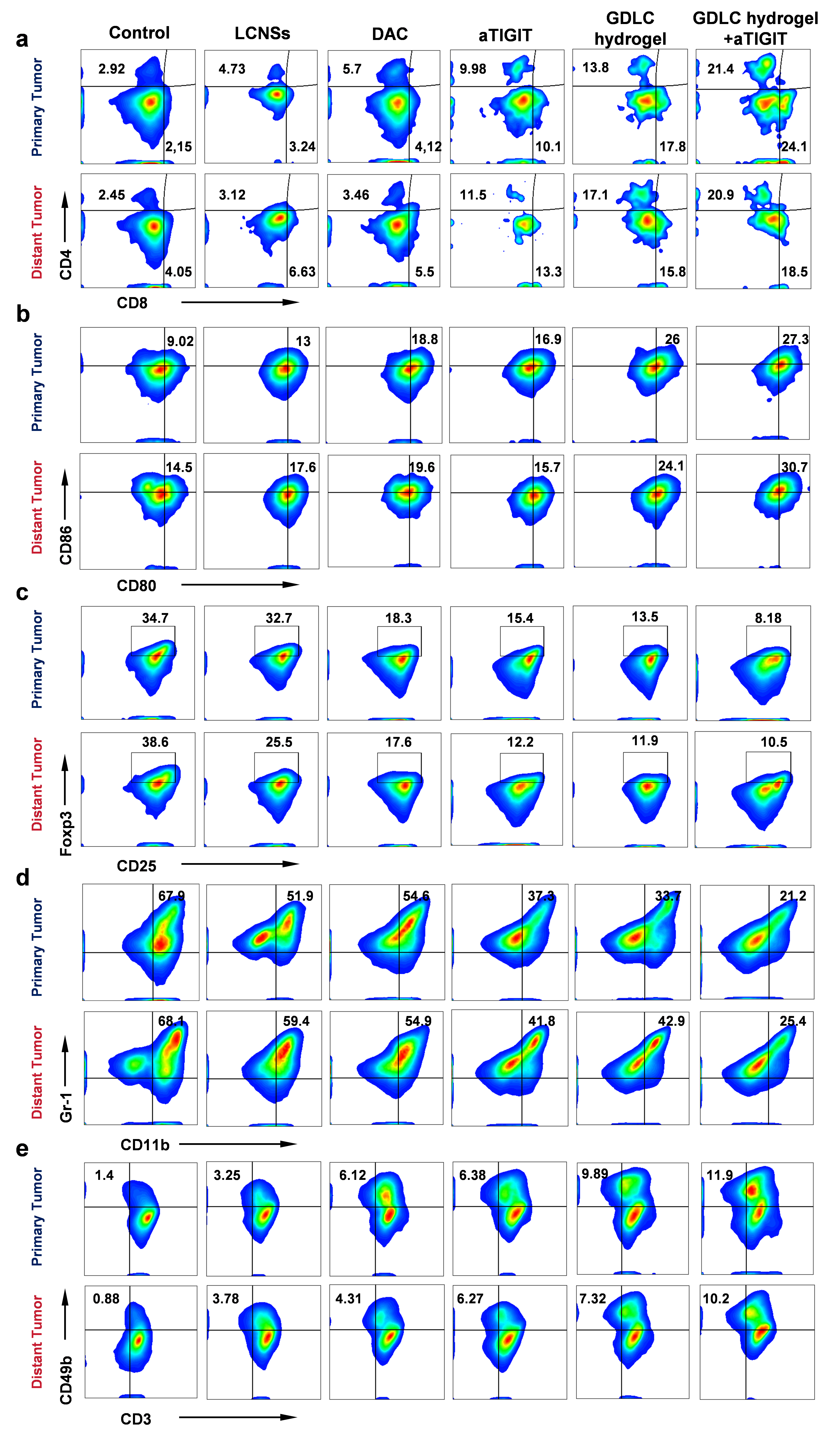


**Figure S27.** Flow cytometric analysis of immune activation in a double tumor model. Flow cytometric analysis of (a) CD4^+^ and CD8^+^ T cells, (b) DCs, (c) Tregs, (d) MDSC, and (e) NK cells in the primary and distant tumors of 4T1-tumor-bearing mice in different treatment groups.


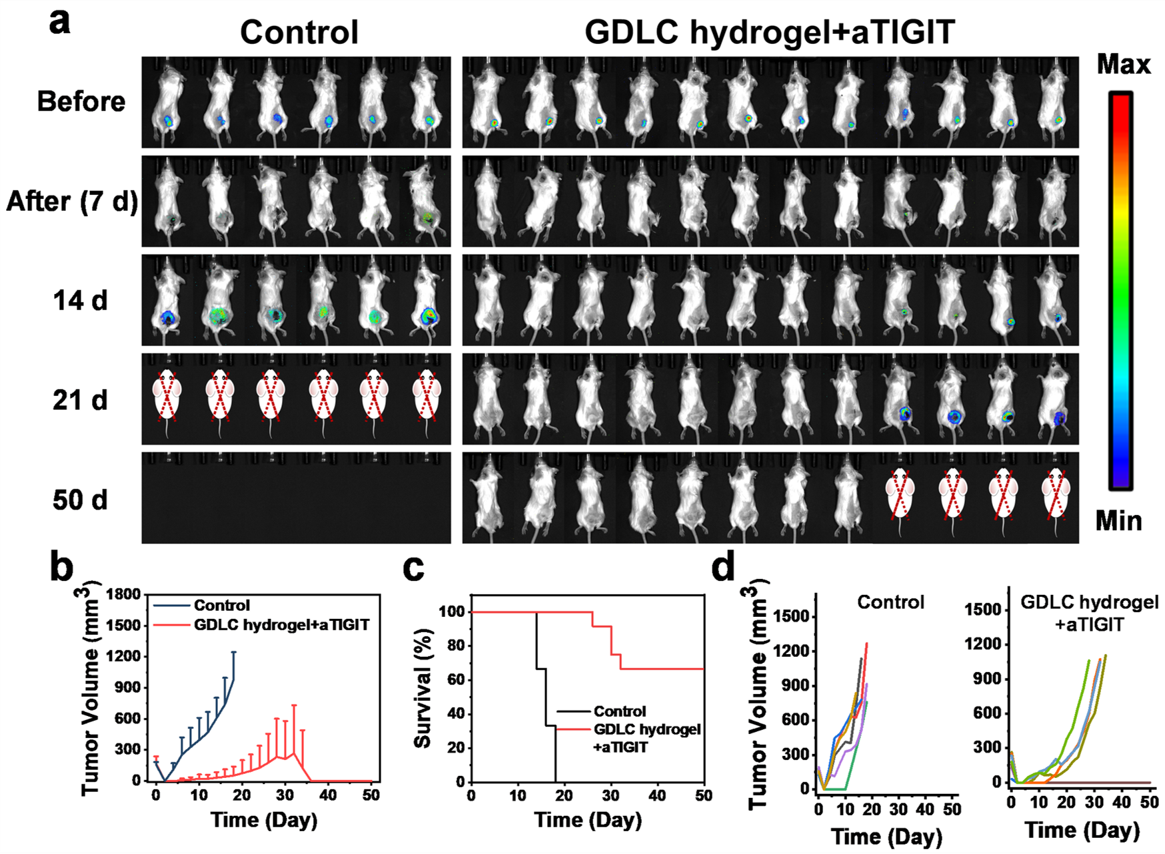


**Figure S28.** Preparation of rechallenged mice in a long-term immune memory model. (a) Bioluminescence images, (b) mean tumor volume, (c) survival rates, and (d) individual tumor growth curves of postoperative mice in the control (*n*=6) and GDLC + aTIGIT group (*n*=12).

**
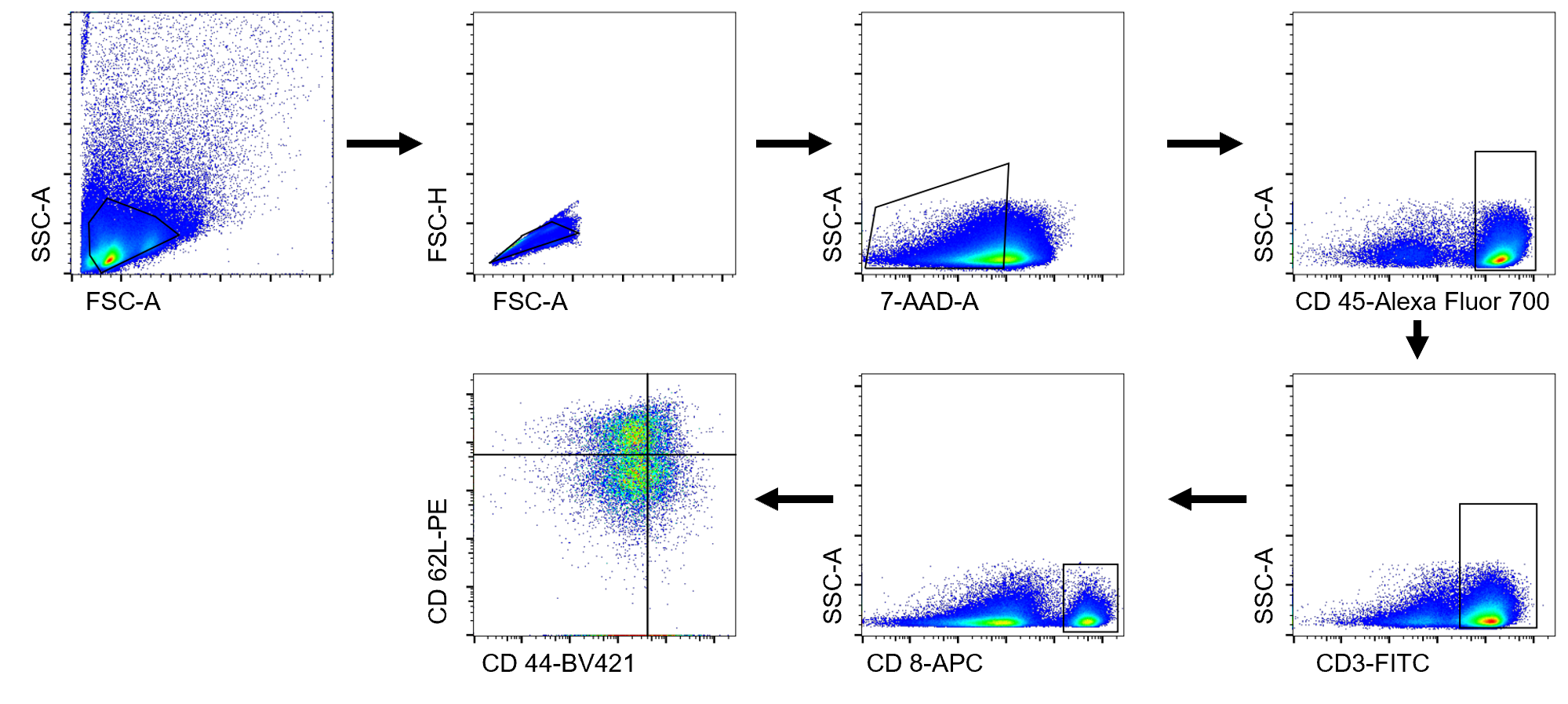
**

**Figure S29.** Flow cytometry gating strategies for T_CM_ and T_EM_ in lymph nodes and spleen.
